# TLR9 signalling in microglia attenuates seizure-induced aberrant neurogenesis in the adult hippocampus

**DOI:** 10.1038/ncomms7514

**Published:** 2015-03-09

**Authors:** Taito Matsuda, Naoya Murao, Yuki Katano, Berry Juliandi, Jun Kohyama, Shizuo Akira, Taro Kawai, Kinichi Nakashima

**Affiliations:** 1 Department of Stem Cell Biology and Medicine, Graduate School of Medical Sciences, Kyushu University, 3-1-1 Maidashi, Higashi-ku, Fukuoka 812-8582, Japan; 2 Laboratory of Gene Regulation Research, Graduate School of Biological Sciences, Nara Institute of Science and Technology (NAIST), Nara 630-0192, Japan; 3 Department of Biology, Bogor Agricultural University, Bogor 16144, Indonesia; 4 Department of Physiology, School of Medicine, Keio University, 35 Shinanomachi, Shinjuku-ku, Tokyo 160-8582, Japan; 5 Laboratory of Host Defense, World Premier International Immunology Frontier Research Center, Osaka University, Osaka 565-0871, Japan; 6 Department of Host Defense, Research Institute for Microbial Diseases, Osaka University, Osaka 565-0871, Japan; 7 Laboratory of Molecular Immunobiology, Graduate School of Biological Sciences, NAIST, Nara 630-0192, Japan

## Abstract

Pathological conditions such as epilepsy cause misregulation of adult neural stem/progenitor populations in the adult hippocampus in mice, and the resulting abnormal neurogenesis leads to impairment in learning and memory. However, how animals cope with abnormal neurogenesis remains unknown. Here we show that microglia in the mouse hippocampus attenuate convulsive seizure-mediated aberrant neurogenesis through the activation of Toll-like receptor 9 (TLR9), an innate immune sensor known to recognize microbial DNA and trigger inflammatory responses. We found that microglia sense self-DNA from degenerating neurons following seizure, and secrete tumour necrosis factor-α, resulting in attenuation of aberrant neurogenesis. Furthermore, TLR9 deficiency exacerbated seizure-induced cognitive decline and recurrent seizure severity. Our findings thus suggest the existence of bidirectional communication between the innate immune and nervous systems for the maintenance of adult brain integrity.

## Abstract (web summary)

Epileptic seizures generate aberrant neurogenesis in the adult mouse hippocampal region but how animals cope with abnormal neurogenesis remains unknown. Here the authors show that microglia are activated through TLR9 signaling and that this leads to sustained expression of TNF-α which attenuates induced aberrant neurogenesis.

## Graphical abstract

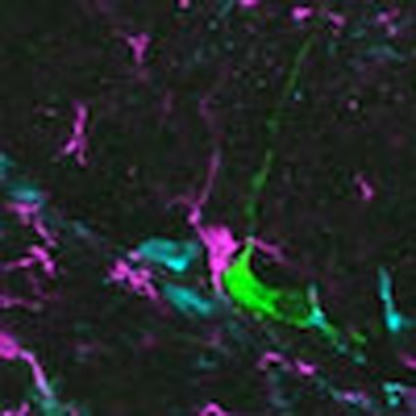

Adult neural stem/progenitor cells (aNS/PCs) in the subgranular zone (SGZ) of the adult hippocampal dentate gyrus (DG) proliferate and give rise to new neurons continuously throughout life to maintain proper brain functions^1^. Although this homoeostatic neurogenesis is strictly controlled under normal physiological conditions, misregulation of aNS/PCs leads to aberrant neurogenesis and impairment of hippocampal-dependent learning and memory under pathological conditions such as stress, depression, ischaemia and epilepsy^2^. The aNS/PC niche, a microenvironment comprising various components including blood vessels, neurons, astrocytes and microglia, is known to contribute to different aspects of neurogenesis under both normal and pathological conditions^2^,^3^,^4^,^5^,^6^,^7^. However, how it responds to pathological conditions to rectify any aberrant behaviour of aNS/PCs is yet to be elucidated.

Microglia, the major immune cell type in the brain, remove dying cells and cellular debris without inducing inflammation under physiological conditions^8^. In response to pathological insults such as infection or brain injury, activated microglia accumulate in the injured site and secrete pro- and/or anti-inflammatory cytokines^9^,^10^. In addition to these functions, increasing evidence suggests that microglia play important roles in aNS/PC regulation under physiological conditions^11^,^12^,^13^.

Toll-like receptors (TLRs) are innate immune receptors that recognize pathogen- or damage-associated molecular patterns (P/DAMPs)^14^,^15^,^16^, and provide an important machinery by which microglia can sense both pathogen- and host-derived ligands and consequently secrete pro- and/or anti-inflammatory cytokines. While TLR2 and TLR4 have been implicated in adult hippocampal neurogenesis under physiological conditions^17^, it is completely unknown whether the nucleic acid-sensing TLR7 and TLR9 can also regulate neurogenesis. To elucidate the functions of these TLRs is important, particularly in pathological conditions, because nucleic acids released from endogenous damaged cells could activate them, resulting in the misregulation of aNS/PC behaviour.

We report here that the loss of TLR9 but not TLR7 reduced seizure-mediated sustained microglial activation and tumour necrosis factor-alpha (TNF-α) production in the hippocampus. Pharmacological inhibition of microglial activation or TNF-α production in wild-type (WT) mice exacerbated aberrant neurogenesis to a similar extent to that observed in TLR9 knockout (KO) mice after seizure. Furthermore, conditioned medium (CM) from hyperactivated hippocampal neurons upregulated *Tnf-α* expression in primary cultures of WT but not TLR9 KO microglia, and this effect was completely abolished when the CM was pretreated with DNase. These results indicate that microglia are activated through TLR9 signalling triggered by self-DNA derived from seizure-induced degenerating neurons in the hippocampus, leading to a sustained expression of TNF-α which attenuates the induced aberrant neurogenesis.

## Results

### TLR9 deficiency aggravates aberrant neurogenesis

We found that Iba1-positive microglia were in close proximity to about 80% of aNS/PCs (positive both for green fluorescent protein (GFP) expressed under the promoter of the NS/PC marker gene *Sox2* and for the aNS/PC marker glial fibrillary acidic protein (GFAP)) in the SGZ of the hippocampus under both physiological and pathological conditions (Supplementary Fig. 1a–c), implying that niche-resident microglia activated in response to pathological insults also affect the behaviour of aNS/PCs by producing pro- and/or anti-inflammatory cytokines.

**Figure 1:**
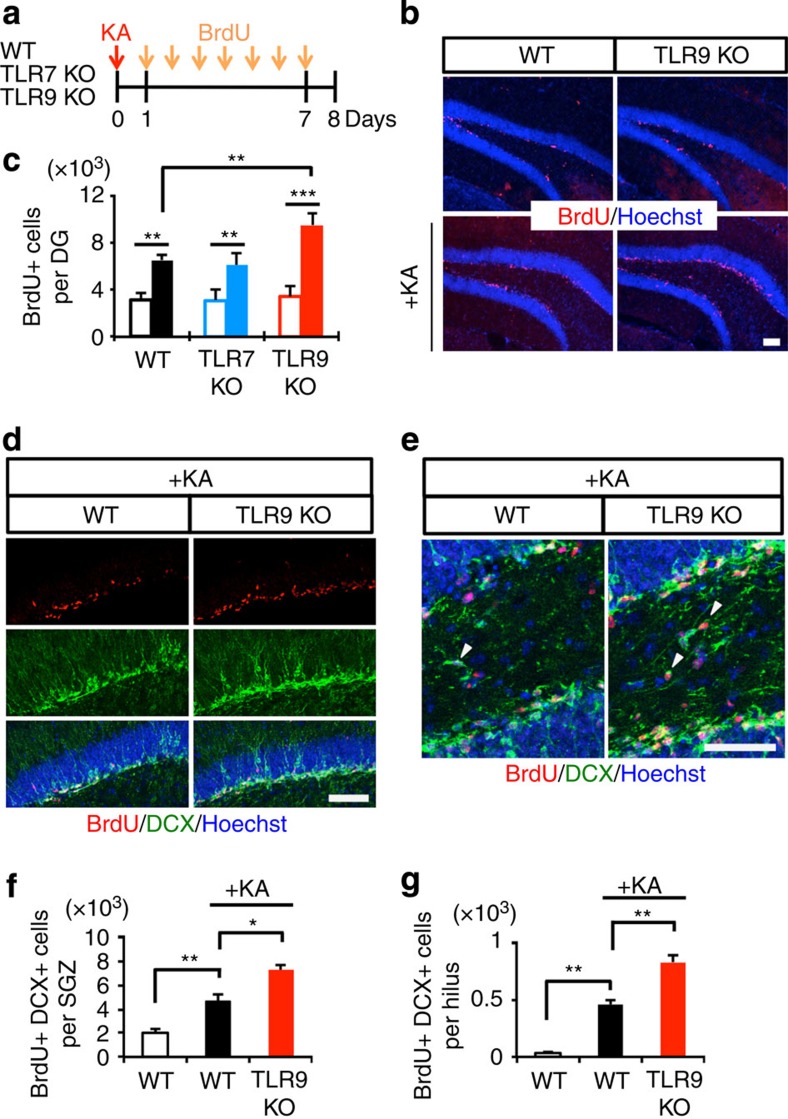
TLR9 deficiency aggravates seizure-induced aberrant neurogenesis. (**a**) Experimental timeline for assessing aNS/PC proliferation in WT, TLR7-KO and TLR9-KO mice. (**b**) Representative images of the DG and stained with BrdU (red) and Hoechst (blue) showed that KA-induced proliferation of aNS/PCs in TLR9-KO mice was more extensive than in WT mice. Scale bar, 50 μm. (**c**) Quantification of total number of BrdU-positive (BrdU+) cells in the DG with (filled bars) or without (open bars) KA treatment. After seizure, the number of BrdU+ cells was higher in TLR9-KO mice than in WT mice (*n*=5 animals). (**d**,**e**) Representative images of BrdU (red) and DCX (green) double-labelled (BrdU+DCX+) newly generated neurons in the SGZ (**d**) and the hilus (**e**) (*n*=5 animals). Scale bars, 50 μm. White arrowheads indicate new neurons located ectopically in the hilus. (**f**,**g**) Quantification of the number of BrdU+DCX+ cells in **c** (SGZ, **f**) and **e** (hilus, **g**; *n*=5 animals). *P<0.05, **P<0.01 and ***P<0.001 by analysis of variance with Tukey *post-hoc* tests.

As a first step towards understanding the roles of TLR7 and TLR9, we confirmed their expression in microglia and found that they are much more highly expressed in these cells than in other neural cell types including NSCs (Supplementary Fig. 1d). To investigate the role of TLR7 and TLR9 in adult hippocampal neurogenesis, we injected bromodeoxyuridine (BrdU) once a day for 7 days into physiologically normal 8-week-old WT, TLR7-KO and TLR9-KO mice to label proliferating aNS/PCs in the DG and killed the mice 1 day after the final injection. We observed no significant difference among these mice (Fig. 1a–c), since TLR signalling is activated by P/DAMPs only under pathological conditions^15^,^16^. Although we have previously reported that convulsive seizure induces aberrant neurogenesis in the adult DG^18^, how animals respond to this pathological condition remains unknown. We thus sought to examine the effects of TLR7 and TLR9 deficiency on the response to convulsive seizure induction. To induce the seizure, we administered kainic acid (KA), a potent glutamate analogue that triggers neuronal hyperactivation, intraperitoneally to WT, TLR7-KO and TLR9-KO mice. KA-induced acute convulsive seizure is known to trigger aberrant augmentation of neurogenesis in the DG and the migration of newborn neurons to ectopic locations such as the hilus, resulting in the impairment of hippocampus-dependent memory^3^,^18^,^19^. Consistent with previous reports, we observed a significant increase in the number of BrdU-positive cells within the SGZ in all KA-treated mice (Fig. 1a–c). Interestingly, while the number of BrdU-positive cells within the SGZ at 1 week after KA administration did not differ between WT and TLR7-KO mice, the number was higher in TLR9-KO mice (Fig. 1a–c). In addition, an increased number of BrdU-retaining and doublecortin (DCX, an immature neuron marker)-expressing newly generated and immature neurons was observed in the DG, including both the SGZ and the hilus, of TLR9-KO mice (Fig. 1d–g), suggesting that the loss of TLR9 stimulates KA-induced aberrant neurogenesis. We decided to focus on TLR9-KO mice in the following experiments because neurogenesis in TLR7-KO mice was indistinguishable from that in WT mice even in the pathological condition.

**Figure 2:**
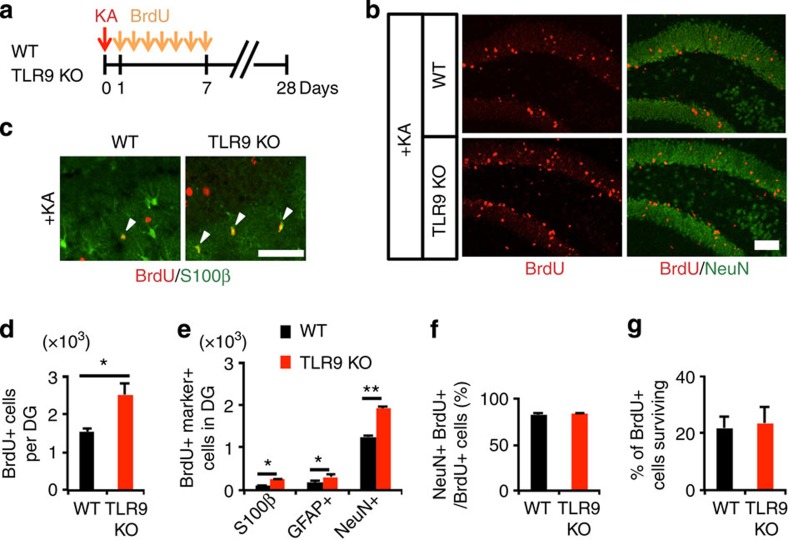
Loss of TLR9 increases newly generated mature neurons after seizure. (**a**) Experimental scheme for examining the number of newly generated mature neurons. (**b**) Representative images of staining for NeuN (green) and BrdU (red) in the DG (*n*=5 animals). Scale bar, 50 μm. (**c**) Representative images of BrdU (red) and S100β (green) staining in the DG of KA-administered WT and TLR9-KO mice. White arrowheads indicate BrdU and S100β double-labelled newborn astrocytes. Scale bar, 50 μm. (**d**,**e**) The DG of TLR9 KO mice exhibited increased numbers of BrdU+ cells (**d**) and newly generated BrdU+NeuN+ mature neurons (**e**) (*n*=5 animals). (**f**,**g**) Quantification of BrdU+ cells for assessing differentiation (**f**) and survival (**g**) of newly generated cells in the DG (*n*=5 animals). (**d**) *P<0.05 by Student's *t*-test. (**e**) *P<0.05 and **P<0.01 by analysis of variance with Tukey *post*-*hoc* tests.

We further traced the differentiation and survival of newly generated cells in the DG at 3 weeks after the last BrdU injection (Fig. 2a). The TLR9-KO mice showed a higher number of BrdU-retaining cells in the DG than that in WT mice (Fig. 2b,d), and the majority of BrdU-retaining cells had become positive for the mature neuronal marker NeuN (Fig. 2b–e). However, the loss of TLR9 had no effect on the proportion of NeuN-positive neurons among BrdU-positive cells. Concordant with the increase in BrdU-positive cells, the number of newly generated S100β- or GFAP-positive astrocytes also increased in the DG of TLR9-KO compared with WT mice (Fig. 2b–e), suggesting that aNS/PC differentiation *per se* was not affected by the loss of TLR9 as shown in Fig. 2f for neuronal differentiation as an example. Furthermore, we measured the proportion of surviving total BrdU-positive cells in WT and TLR9-KO mice. To calculate the ratio of surviving cells after seizure, we divided the total number of BrdU-positive cells at 3 weeks after the last BrdU injection by that at 1 day after the last BrdU injection. We found no difference in the survival ratio (Fig. 2g), indicating that TLR9 signalling does not contribute to the survival of newborn cells in the DG. Taken together, these results suggest that TLR9 deficiency aggravates seizure-induced aberrant neurogenesis in the hippocampus by promoting aNS/PC proliferation. In other words, these data indicate that TLR9 signalling attenuates seizure-induced abnormal proliferation of aNS/PCs to maintain homoeostatic neurogenesis in the DG.

### Activated microglia attenuates aberrant neurogenesis

Before further analyzing TLR9 function, we determined whether TLR9 is indeed expressed in microglia in the DG *in vivo*. As shown in Supplementary Fig. 1e, Iba1-positive microglia clearly expressed TLR9. We also examined whether i.p. KA injection compromises the integrity of the blood–brain barrier (BBB) by using Evans blue dye, because macrophages infiltrate the damaged brain in conditions such as ischaemia through a disrupted BBB. However, Evans blue dye leakage was not found in the brain, while the dye was detected in other organs in both WT and TLR9-KO mice, indicating that the BBB remains intact after KA injection in these mice (Supplementary Fig. 1f). Although we cannot completely exclude the possibility that macrophages infiltrate the brain after seizure through the intact BBB, any such macrophage population would be exceedingly small compared with the brain-resident microglia. We therefore focused mainly on microglia as TLR9-expressing cells in this study.

**Figure 3:**
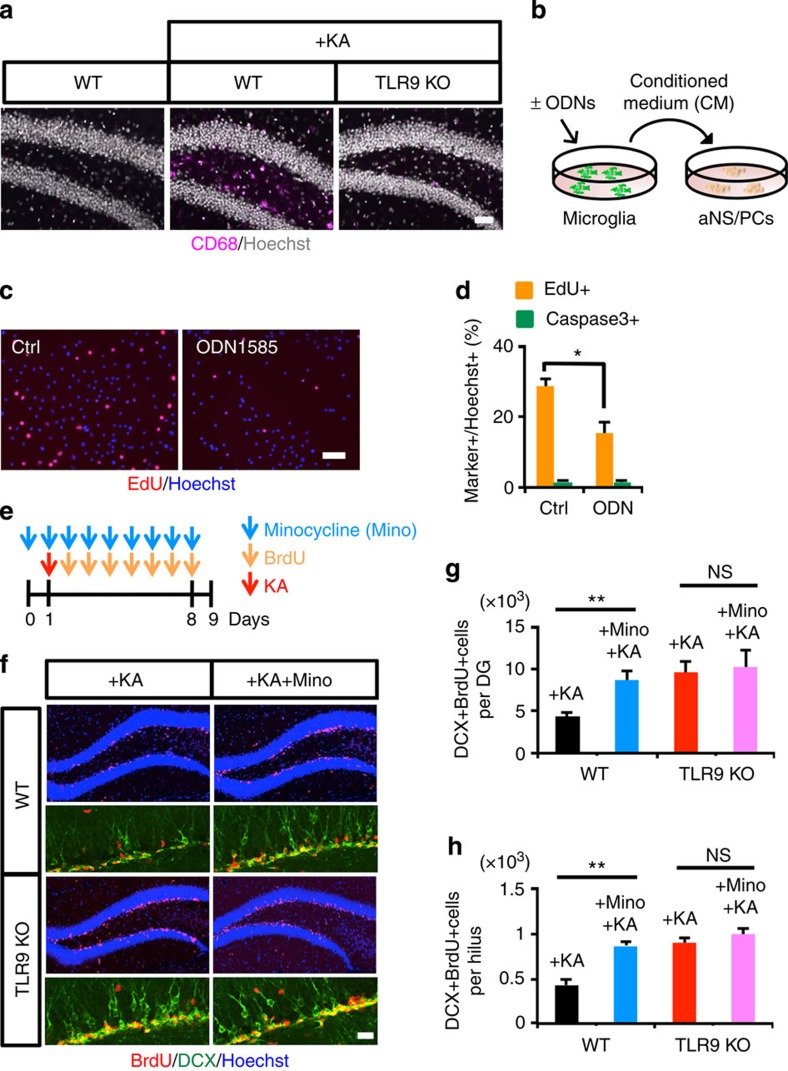
Activated microglia attenuates aberrant neurogenesis. (**a**) Representative images of CD68+ (magenta) activated microglia in the DG of TLR9 KO and WT mice with or without KA treatment (*n*=4 animals). Scale bar, 50 μm. (**b**) Experimental scheme for assessing aNS/PC proliferation in the presence or absence of CM derived from ODN1585-stimulated microglia. (**c**) Representative images of EdU (red) and Hoechst (blue) staining of aNS/PCs cultured with CM of ODN1585-treated (right) and untreated (left) microglia as a control (Ctrl) (*n*=5 experiments). Scale bar, 50 μm. (**d**) Quantification of EdU+Hoechst+ cells or an apoptotic cell marker active caspase3+Hoechst+ cells. ODN1585-dependent microglial activation inhibits aNS/PCs without affecting cell survival (*n*=5 experiments). (**e**) Experimental scheme for assessing aNS/PC proliferation in minocycline-treated mice. (**f**) Representative images of BrdU+ (red) DCX+ (green) newly generated neurons in the DG. Scale bar, 50 μm. (**g**,**h**) Quantification of the number of BrdU+DCX+ cells in the DG (**g**) and hilus (**h**) (*n*=5 animals). Ectopic neurogenesis increased in minocycline-treated mice to a similar extent to that observed in TLR9 KO mice (*n*=5 animals). (**d**) *P<0.01 by Student's *t*-test. (**g**,**h**) NS means not significant (*P*>0.05). *P<0.05, **P<0.01 by analysis of variance with Tukey *post-hoc* tests.

Since it has been shown that epileptic seizure induces microglial activation in the hippocampus^20^, we evaluated the microglial activation status in WT and TLR9-KO DG after seizure by immunohistochemistry with an antibody against CD68, which is upregulated in activated microglia (Supplementary Fig. 2a,b). Many CD68-positive activated microglia were detected in WT mice 7 days after seizure, whereas the activation was much lower in TLR9-KO mice, indicating that sustained microglial activation in the DG requires functional TLR9 (Fig. 3a). This result prompted us to ask whether TLR9-mediated microglial activation inhibits aNS/PC proliferation. To induce microglial activation, we prepared primary cultured microglia (Supplementary Fig. 3a) and stimulated them *in vitro* with the TLR9 ligand ODN1585. Incubation of microglia with ODN1585 upregulated the expression of two well-known targets of TLR signalling, *Tnf-α* and *Interferon* (*Ifn*) β, in a dose-dependent manner (Supplementary Fig. 3b). We then tested whether the CM of microglia activated with ODN1585 affects aNS/PC proliferation, and found that it decreased aNS/PC proliferation compared with control CM without affecting cell survival (Fig. 3b–d). We also performed experiments using different ligands (ODN2395 and ODN1826) for TLR9, and found that CM from microglia activated with these two ligands likewise inhibited aNS/PC proliferation (Supplementary Fig. 4). These data indicate that microglia activated through TLR9 inhibit aNS/PC proliferation *in vitro*.

Minocycline, a semisynthetic tetracycline derivative, is known to inhibit microglial activation *in vivo*^21^. To inhibit sustained microglial activation after seizure, we injected minocycline intraperitoneally into WT and TLR9-KO mice once daily for 8 consecutive days. We confirmed that seizure-induced microglial activation was inhibited by minocycline treatment (Supplementary Fig. 2b). When we inhibited microglial activation with minocycline in KA-administered WT mice, seizure-induced aberrant neurogenesis was exacerbated, reaching a level similar to that observed in TLR9*-*KO mice treated with KA alone (Fig. 3e–h). Furthermore, there were no differences in the number of BrdU- and DCX-positive cells (newly generated neurons) between minocycline-treated or -untreated TLR9-KO mice after seizure (Fig. 3f–h). Taken together, these results suggest that activated microglia attenuate seizure-induced neurogenesis through TLR9 signalling. Minocycline itself did not affect neurogenesis under normal physiological conditions (Supplementary Fig. 2c–e).

### Microglia-derived TNF-α alleviates aberrant neurogenesis

**Figure 4:**
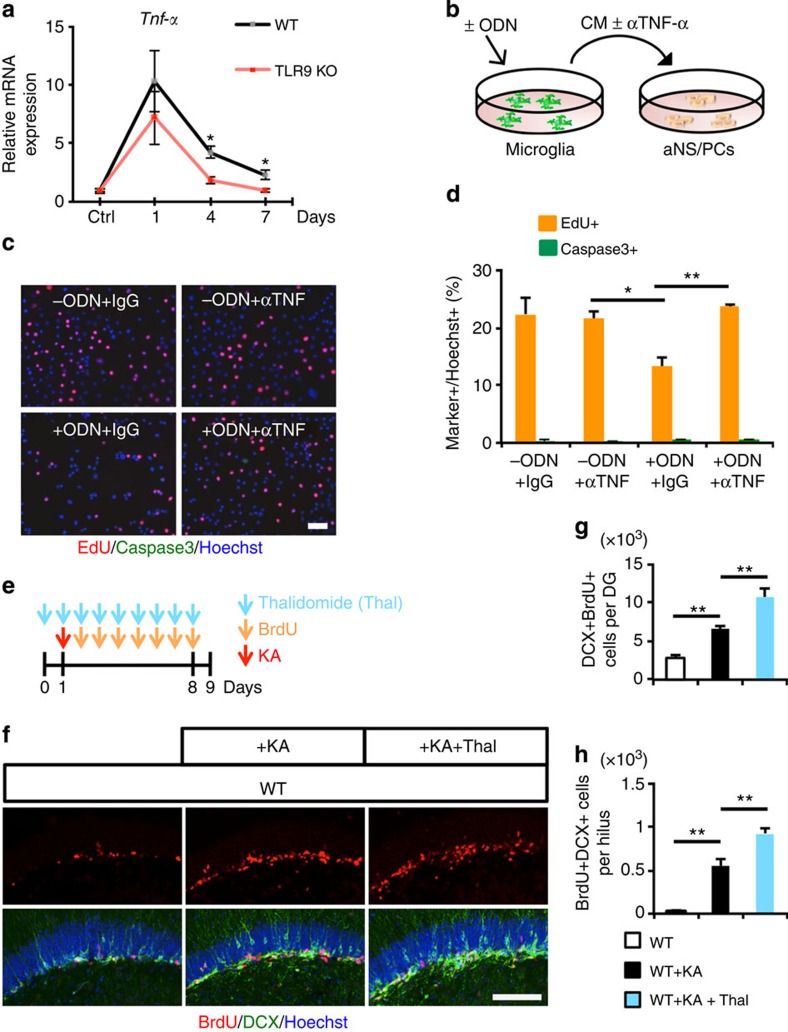
Microglia-derived TNF-α alleviates aberrant neurogenesis. (**a**) qRT-PCR analyses of *Tnf-α* levels in the DG of WT and TLR9 KO mice at the indicated time points after seizure. Experimental controls were KA-untreated WT and TLR9 KO mice, respectively (*n*=3 animals). *P<0.05 by analysis of variance (ANOVA) with Tukey *post-hoc* tests. (**b**) Experimental scheme for assessing aNS/PC proliferation in microglia culture-derived CM pretreated with TNF-α-neutralizing antibody or IgG control. (**c**) Representative images of EdU (red), active caspase3 (green) and Hoechst (blue) staining in aNS/PCs cultured with the indicated CM from microglia (*n*=4 experiments). Scale bar, 50 μm. (**d**) Quantification of EdU+ or active caspase3+ cells in **c** (*n*=4 experiments). (**e**) Experimental timeline for assessing aNS/PC proliferation in thalidomide (Thal)-treated mice. (**f**) Representative images of BrdU+DCX+ newly generated immature neurons in the DG (*n*=4 animals). Scale bar, 50 μm. (**g**,**h**) Quantification of the number of BrdU+DCX+ cells in **f**, in the DG (**g**) and in the hilus (**h**) (*n*=4 animals). *P<0.05 and **P<0.01 by ANOVA with Tukey *post-hoc* tests.

Recent studies have shown that various inflammation-related molecules regulate adult neurogenesis in the DG^22^,^23^,^24^, leading us to hypothesize that such molecules released from activated microglia might attenuate aberrant neurogenesis induced by seizure. To test this, we performed quantitative real-time PCR (qRT–PCR) analysis. At one day after seizure induction, expression of inflammatory cytokines, such as *Il-1β*, *Il-6* and *Tnf-α*, in both WT and TLR9-KO DG was upregulated compared to KA-untreated controls (Fig. 4a and Supplementary Fig. 5). Intriguingly, the higher expression level of *Tnf-α* and not other mRNAs was sustained in KA-treated WT mice at 4 or 7 days after seizure, yet the *Tnf-α* expression level in TLR9-KO mice reverted to the control level by 4 days after seizure induction (Fig. 4a). Given that TNF-α has previously been shown to suppress neurogenesis in the DG^23^, we tested whether TNF-α inhibits aNS/PC proliferation *in vitro*, and found that it did so in a dose-dependent manner (Supplementary Fig. 6a,b). We also examined whether IL-12 and IFN-γ inhibit aNS/PC proliferation, because their expression differed between WT and TLR9-KO mice at day 1 after seizure (Supplementary Fig. 5). However, we found no difference in the number of EdU (a thymidine analogue)-positive cells irrespective of IL-12 and IFN-γ treatment, suggesting that aNS/PC proliferation is unaffected by these cytokines (Supplementary Fig. 6c–f). Therefore, we decided to focus on TNF-α in subsequent experiments. To examine whether TLR9 signalling-mediated TNF-α production by microglia inhibits aNS/PC proliferation, we pretreated CM samples from microglia with a TNF-α-neutralizing antibody or immunoglobulin-G (IgG) control, and added them to aNS/PC cultures (Fig. 4b). ODN1585-untreated microglial CM pretreated with the TNF-α antibody did not affect aNS/PC proliferation. In marked contrast, the anti-proliferative effect of the CM from ODN1585-treated microglia was abolished with the TNF-α antibody, indicating that TLR9 stimulation-induced TNF-α is the factor responsible for the inhibition of aNS/PC proliferation (Fig. 4c,d).

Thalidomide is known as a BBB-permeable inhibitor of TNF-α production^25^. To allow administered thalidomide enough time to be fully effective in inhibiting *Tnf-α* expression by the time of KA injection, we started the injection of thalidomide into WT mice 1 day before the KA injection, and continued once daily until 3 days after seizure; the mice were then killed 4 days after seizure. As shown in Supplementary Fig. 6g,h, we found that thalidomide treatment suppressed *Tnf-α* expression in the DG of WT mice 4 days after seizure. When we injected thalidomide into WT mice once daily for 8 consecutive days, in accordance with our *in vitro* experiments, suppression of TNF-α production aggravated aberrant neurogenesis *in vivo* as observed in thalidomide-untreated TLR9-KO mice after seizure (Fig. 4e–h).

Next, to understand more precisely the behaviour of aNS/PCs in response to seizure, we examined aNS/PC proliferation at earlier time points after KA administration in both WT and TLR9-KO mice. After 1, 2, 3 and 4 days of KA administration, we injected BrdU into these mice every 4 h (four times) and killed them 12 h after the last BrdU injection (Supplementary Fig. 7a). We found that the number of BrdU-positive cells which were proliferating at day 4 after seizure induction in WT mice was significantly higher than in untreated WT mice (Supplementary Fig. 7b,d). In contrast, statistically different BrdU-positive cell numbers between untreated and KA-treated mice were already observed at day 2 in TLR9-KO mice (Supplementary Fig. 7b,d). Moreover, these trends of increased BrdU-positive cell numbers in WT and TLR9-KO mice were inversely correlated with the degree of microglial activation and *Tnf-α* expression in these mice (Supplementary Fig. 7c,e). As shown in Supplementary Fig. 7e, a dramatic upregulation of *Tnf-α* expression in both WT and KO mice was observed at day 1 after KA injection, probably attributable to a direct effect of KA. By 2 days, *Tnf-α* expression in KA-injected TLR9-KO mice had already reverted to a level similar to that in the control, but was still high in KA-injected WT mice, suggesting that the difference was attributable to TLR9 deficiency. To confirm the effectiveness of thalidomide on the inhibition of *Tnf-α* expression, we then injected thalidomide into WT mice 1 day before the difference in *Tnf-α* expression between WT and TLR9 KO mice became apparent (day 2) (Supplementary Fig. 8a). As shown in Supplementary Figs 7d and 8c, BrdU-positive cells had not yet increased in response to KA treatment at day 3 in WT mice, but we did observe an increase of BrdU-positive cells in response to thalidomide treatment (Supplementary Fig. 8b,c). We then examined whether TNF-α reduces exacerbated neurogenesis in TLR9-KO mice after seizure. We infused recombinant TNF-α protein into the DG of TLR9-KO mice after seizure (Supplementary Fig. 9a) and found that TNF-α indeed reduced the seizure-induced aberrant neurogenesis in these mice (Supplementary Fig. 9b,c). Taken together, these results indicate that TLR9 signal-regulated TNF-α production by microglia is a critical process to withstand the aberrant neurogenesis following seizure.

### TLR9 signalling does not affect localization of new neurons

Having shown that TLR9-dependent TNF-α production in microglia attenuates seizure-induced aNS/PC proliferation and ectopic generation of new neurons, we next asked whether TLR9 signalling affects the location of DCX-positive newly generated neurons in the DG at 8 days after seizure. The loss of TLR9 had no effect on the distribution of DCX-positive neurons, whereas the number of DCX-positive neurons in each area examined was increased in TLR9-KO mice after seizure compared with WT mice (Supplementary Fig. 10a–c). Since microglia activation and TNF-α production are important processes downstream of TLR9 signalling, minocycline and thalidomide treatment did not affect the distribution but did increase the number of DCX-positive cells, similar to TLR9 loss (Supplementary Fig. 10b). We also examined the morphology of DCX-positive cells after seizure by Sholl analysis and measurement of dendrite length. TLR9-KO mice showed no significant differences in these analyses, although KA itself affected the morphology of DCX-positive neurons (Supplementary Fig. 11a–c). Thus, these data indicate that TLR9 deficiency aggravates seizure-indu**c**ed aberrant neurogenesis in the hippocampus by promoting aNS/PC proliferation.

### Loss of TLR9 worsens seizure-induced behavioural impairments

**Figure 5:**
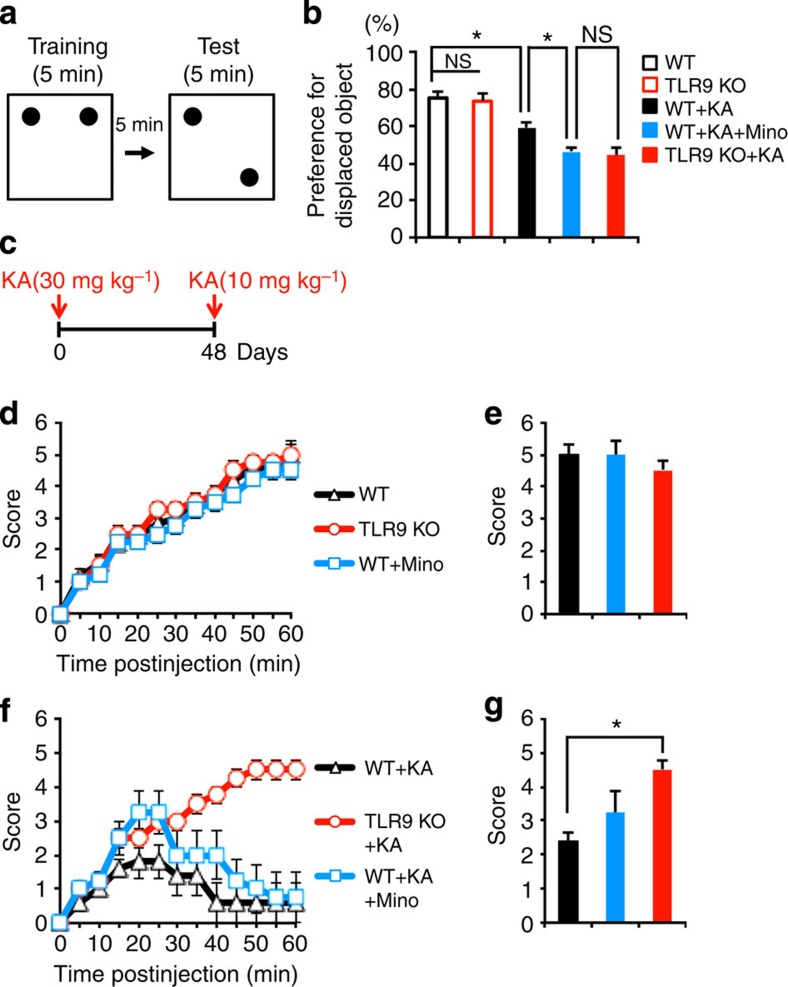
TLR9 deficiency worsens seizure-induced behavioural impairments. (**a**) Schematic paradigm of place recognition test. Five minutes after training phase, mice were allowed to explore two objects one of which was displaced to different location from the original location. (**b**) KA-treated WT mice did not show much preference for the displaced object compared with untreated WT mice. This cognitive decline was exacerbated in TLR9 KO mice. KA-treated WT mice that received minocycline treatment showed the similar level of preference for the displaced object as KA-treated TLR9-KO mice did. Each group has eight mice. NS means not significant (*P*>0.05). *P<0.05 by analysis of variance (ANOVA) with Tukey *post-hoc* tests. (**c**) Experimental scheme for examining recurrent seizure severity. All mice were administrated with KA (10 mg kg^−1^) 48 days after first KA injection (30 mg kg^−1^). (**d**) Seizure response over time in WT, TLR9 KO and minocycline-treated WT mice following the first KA injection (30 mg kg^−1^) (*n*=8 animals). For each 5-min interval, the highest level of seizure activity was scored using previously described scale (see Methods). (**e**) The maximum score of each mouse group during the 60-min trial in **d**. (**f**) Seizure response over time in WT mice, TLR9 KO mice and minocycline-treated WT mice following KA injection (10 mg kg^−1^) 48 days after first KA injection (*n*=8 animals). (**g**) The maximum score of each mouse group during the 60-min trial in **f**. *P<0.01 by ANOVA with Tukey *post-hoc* tests.

We have previously shown that seizure-induced aberrant neurogenesis impairs a hippocampal-dependent cognitive function^18^. Since seizure-induced aberrant neurogenesis is exacerbated in TLR9-KO mice, we examined whether KA-treated TLR9-KO mice show further cognitive decline by subjecting KA-treated or -untreated WT and TLR9-KO mice to a hippocampus-dependent place recognition task (Fig. 5a). Administration of KA to WT mice induced cognitive decline in agreement with our previous report^18^ (Fig. 5b). Moreover, the loss of TLR9 aggravated this seizure-induced cognitive decline, although without KA treatment WT and TLR9-KO mice showed no significant difference in the test (Fig. 5b). When we inhibited microglial activation in WT mice treated with KA using minocycline, the level of impairment in these mice decreased to an extent similar to that observed in TLR9-KO mice without treatment (Fig. 5b). These data suggest that TLR9-mediated microglial activation attenuates seizure-induced cognitive decline in WT mice.

A recent study has indicated that ectopically located new neurons contribute to epileptogenesis^26^. This observation prompted us to examine whether either TLR9 deficiency or inhibition of microglia activation by minocycline affect the severity of seizure induced by KA re-injection at 48 days after the first KA injection (Fig. 5c). All mice showed no difference in scores of seizure following the first KA injection (Fig. 5d,e). Furthermore, the first administration of KA at low concentration did not affect the scores in WT or TLR9-KO mice (Supplementary Fig. 12a,b). However, when we re-injected KA at low concentration to TLR9-KO mice 48 days after the first KA injection, they developed more severe seizures compared with WT mice (Fig. 5f,g). Minocycline treatment indeed aggravated the symptoms in WT mice, *albeit* to a lesser extent than that observed in TLR9 KO mice without minocycline, probably because we ceased minocycline treatment at 7 days after the first KA injection. During the 7 days after the first KA administration, microglial activation was inhibited by minocycline, but resumed after the cessation of this treatment. We therefore inferred that the inhibition of aberrant neurogenesis was less effective in minocycline-treated WT mice than in untreated WT mice. Taken together, these results suggest that TLR9 signalling attenuates seizure-induced cognitive decline and recurrent seizure severity.

### Degenerating neuron-derived DNA activates TLR9 signalling

**Figure 6:**
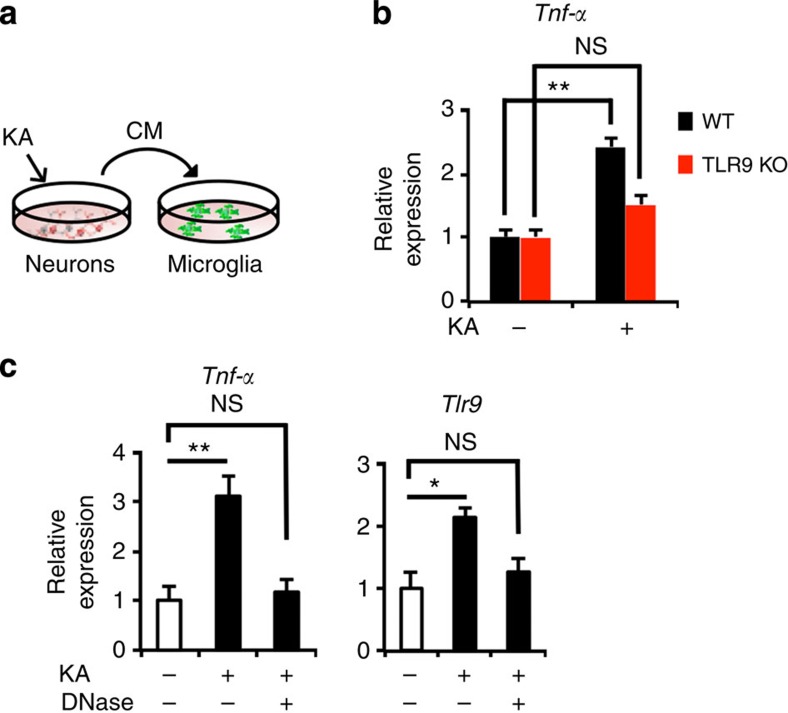
Degenerating neuron-derived DNA activates TLR9 signalling. (**a**) Experimental scheme for the identification of an endogenous ligand for TLR9 expressed in microglia. (**b**) qRT-PCR analyses of *Tnf-α* levels in microglia from WT and TLR9 KO mice 12 h after incubation with CM from KA-treated or -untreated neurons. TLR9 KO microglia failed to induce *Tnf-α* expression in response to CM from KA-treated neurons (*n*=4 experiments). (**c**) qRT-PCR analyses of *Tnf-α* expression levels in microglia from WT mice 12 h after incubation with DNase-pretreated CM from KA-treated neurons (*n*=5 experiments). NS means not significant (*P*>0.05). *P<0.05 and **P<0.01 by ANOVA with Tukey *post-hoc* tests.

Finally, we addressed the possibility that an endogenous cell-derived ligand activates TLR9 signalling in microglia after seizure. Increasing evidence indicates that, in addition to detection of PAMPs, TLRs contribute to the detection of damage to the CNS by recognizing endogenous DAMPs released from dying or degenerating cells^15^,^16^,^27^. As we have shown previously^18^, seizure causes neuronal degeneration in the DG. We collected CM from hippocampal neurons stimulated with KA, and cultured primary microglia in its presence for 12 h (Fig. 6a). We found that the *Tnf-α* expression level in primary WT microglia was increased by the CM of KA-treated neurons. In contrast, the CM failed to induce *Tnf-α* expression in TLR9-KO microglia (Fig. 6b). When we pretreated the CM from KA-treated neurons with DNase, the CM-induced elevation of *Tnf-α* expression in primary microglia was abolished (Fig. 6c). These data suggest that DNA derived from degenerating neurons activates TLR9 signalling in microglia as an endogenous ligand. Moreover, we observed that the CM of KA-treated neurons upregulated the expression of *Tlr9* in primary microglia, indicating the existence of a positive feedback loop to enhance TLR9 signalling in microglia (Fig. 6c). CM-induced *Tlr9* expression was also severely compromised by pretreatment with DNase.

The TLR9 signalling pathway is known to activate NF-κB, which controls the expression of inflammatory cytokine genes^28^. We examined whether CM from degenerating neurons induces NF-κB activation in microglia via TLR9. When we cultured microglia with CM derived from KA-stimulated hippocampal neurons, p65 protein, a subunit of the NF-κB complex, translocated into the nucleus, indicating that the CM can indeed activate NF-κB in microglia (Supplementary Fig. 13a,b). This activation was abolished by DNase or TLR9 inhibitor (ODN2088) treatment (Supplementary Fig. 13a,b). These data suggest that DNA derived from degenerating neurons evokes NF-κB activation in microglia via TLR9.

## Discussion

TLR9 was initially identified as a receptor that recognizes microbial DNA^29^, but current research has shown that TLR9 can sense self-DNA as a DAMP^30^, and it appears to be involved in numerous immune processes and autoimmune diseases^14^. However, it is unknown whether TLR9 senses self-DNA to modulate CNS function in the absence of pathogen-derived DNA. In the present study, we have suggested that TLR9 senses self-DNA from degenerating hippocampal neurons to attenuate seizure-induced aberrant neurogenesis, revealing a role for TLR9 in maintaining brain integrity. TLR7-KO mice, in contrast, displayed no impaired phenotype, at least in neurogenesis. Moreover, when we pretreated CM from KA-treated neurons with RNase to deplete TLR7 ligand (RNA), the CM-induced elevation of *Tnf-α* expression in primary microglia was not abolished (Supplementary Fig. 14). These data suggest that self-RNA from degenerating neurons does not function as a TLR7 ligand for the regulation of neurogenesis following seizure, although TLR7 is known to recognize self-RNA and microRNA^31^,^32^. The microRNA let-7, a highly abundant regulator of gene expression in the CNS, activates TLR7 in neurons and induces neurodegeneration^31^. Neuronal TLR7 is also reported to sense self-RNA derived from neighbouring cells and thus to impair axonal outgrowth of cortical neurons^32^. These observations warrant further experimentation using TLR7-KO and/or a double-KO with TLR9 to gain a better understanding of how immune receptors recognize endogenous ligands from neighbouring CNS cells and modulate brain functions.

We have shown that the activation of TLR9 signalling in microglia induces TNF-α expression, resulting in the attenuation of aberrant neurogenesis in the hippocampus. Consistent with our findings, it has been reported that TNF receptor 1 (TNFR1) expressed in aNS/PCs is a negative regulator for adult hippocampal neurogenesis^23^. The loss of TNFR1 enhances seizure-mediated aNS/PC proliferation and increases neurogenesis in the hippocampus. TNF-α is a key factor in the immune response^33^, but it is unclear whether TNF-α has any positive effects in the CNS since many studies have focused on its roles as a negative effector in neurodegenerative disorders such as Alzheimer's disease^34^. Thus, our results unveil a positive aspect of TNF-α function in the CNS for the maintenance of homoeostatic neurogenesis.

The findings that microglia are in close proximity to aNS/PCs (Supplementary Fig. 1a–c) and that inhibition of microglial activation after seizure exacerbates aberrant neurogenesis (Fig. 3e–h and Supplementary Fig. 2) highlight the importance of microglia for the regulation of adult neurogenesis in the aNS/PC niche. Consistent with these observations, several previous studies have suggested that microglia inhibit adult hippocampal neurogenesis^12^,^13^. However, it has also been suggested that activated microglia promote neurogenesis by secreting trypsinogen^35^. These contradictory assertions are probably due to the contribution of different subtypes of activated microglia to the regulation of neurogenesis. Microglia can undergo different modes of polarized activation, which give rise to potentially neurotoxic classic-M1 (characterized by the release of pro-inflammatory factors) or potentially neuroprotective alternative-M2 (characterized by the expression of anti-inflammatory cytokines) subtypes^10^,^36^,^37^. In the CNS, increasing evidence indicates that M1 microglia exacerbate neurodegenerative disease through the production of pro-inflammatory cytokines^10^. Minocycline is known as an inhibitor of M1 microglial activation since it selectively inhibits M1 microglia-related gene expression^21^. Therefore, it is plausible that M1 microglia release TNF-α and consequently attenuate seizure-induced aberrant neurogenesis.

In this study, we have identified a novel, intrinsic mechanism to attenuate aberrant neurogenesis that involves interactions between TLR9-expressing immune cells and neural cells. We show that TLR9 signalling activated by DNA from degenerating neurons induces TNF-α production in microglia, resulting in the inhibition of seizure-induced aberrant neurogenesis (Supplementary Fig. 15). Thus, microglia in the aNS/PC niche respond via TLR9 to the insult induced by seizure and ensure homoeostatic neurogenesis. However, we suspect that our proposed mechanism is one among many that contribute to homoeostatic neurogenesis, because the aNS/PC niche is composed of many types of cells and is vulnerable to a variety of pathological stimuli. Future studies will reveal as-yet-unknown mechanisms by which the niche reacts to these stimuli to ensure the maintenance of brain homoeostasis. Boosting the functions of these intrinsic mechanisms should offer therapeutic strategies for diseases associated with abnormal neurogenesis, such as major depression, ischaemia, Alzheimer's disease and epilepsy.

## Methods

### Animals

All efforts were made to minimize animal suffering and to reduce the number of animals used. Animals were housed on a 12/12-h light/dark cycle and fed *ad libitum*. TLR7-KO and TLR9-KO mice were on a C57BL/6 background. Sox2–GFP mice^38^ were a gift from F.H. Gage (Salk Institute, USA). Male 8-week-old mice were used for this study. To induce seizure, the mice received i.p. injections of KA (30 mg kg^−1^; Enzo Life Sciences) dissolved in saline. Behaviour of KA-treated mice was observed for 1 h after the injection and the seizure score was recorded, according to previously described criteria^39^,^40^. Briefly, we used the following seizure scale: no response (0), staring and reduced locomotion (1), activation of extensors and rigidity (2), repetitive head and limb movements (3), sustained rearing with clonus (4), loss of posture (5), and status epilepticus and death (6). The scores did not differ between WT and TLR9 KO mice following KA injection. To assess recurrent seizure severity, all mice were re-injected with KA (10 mg kg^−1^) at 48 days after the first KA injection (30 mg kg^−1^).

The day after KA injection, BrdU (50 mg kg^−1^; Sigma) dissolved in saline was injected intraperitoneally into TLR7-KO, TLR9-KO and WT mice daily for 1 week to monitor cell proliferation, differentiation and survival. These mice were killed 1 day or 21 days after the last injection of BrdU. The day before seizure induction, minocycline (20 mg kg^−1^; Sigma) dissolved in saline was injected intraperitoneally into TLR9-KO and WT mice once daily for 8 consecutive days to inhibit microglial activation. To inhibit seizure-induced TNF-α production, thalidomide (250 mg kg^−1^; Sigma) dissolved in 0.5% carboxymethylcellulose (Sigma) was injected intraperitoneally into WT mice with the same time course as minocycline. For the inhibition of TLR9-dependent TNF-α production, mice were treated with thalidomide on day 1 after seizure and again the following day. To evaluate cell proliferation at each day, we injected BrdU into mice every 4 h (four times) at 1, 2, 3 and 4 days after KA administration and killed the mice 12 h after the last BrdU injection.

For the hippocampus-dependent recognition test, mice were transferred to the testing room and acclimated for at least 1 h before habituation and testing. Each mouse was habituated to the empty testing chamber (10 min) for 3 days after being handled for 3 days. The testing chamber was an opaque plastic chamber (50 × 50 × 30 cm). Two identical objects were placed in the testing chamber, and the mouse was allowed to explore the objects for 5 min as the training phase and then the mouse was taken out from the chamber. We then placed one of the two objects in a new position where is a diagonal corners with the object placed in familiar position (Fig. 5a). After a 5-min delay, the mouse was replaced in the testing chamber again. The mouse was given 5 min to explore the familiar and displaced objects during the testing phase. Behaviour was recorded with a video tracking system. Frequency of object interactions and time spent exploring each object were recorded for subsequent data analysis. The testing chamber and used objects were washed with 70% ethanol before the next mouse was tested. All mice were subjected to behavioural testing at 6 weeks after seizure.

All mice were treated according to Fundamental Guidelines for Proper Conduct of Animal Experiment and Related Activities in Academic Research Institutions under the jurisdiction of the Ministry of Education, Culture, Sports, Science and Technology of Japan.

### Gene expression analysis

Total RNA was isolated from tissues and cells using Sepasol-RNA I Super G (Nacalai Tesque) following the manufacturer's instructions. RNA quality of all samples was checked by spectrophotometer. Reverse transcription reactions were carried out using the SuperScript VILO cDNA Synthesis Kit (Life Technologies) according to the kit protocol. Primer sequences used in this study can be found in Supplementary Table 1. qRT–PCR was performed with SYBR green fluorescent dye using Step One Plus (Applied Biosytems) and Mx3000 (Stratagene). GAPDH was used as an endogenous control to normalize samples.

### Immunohistochemistry

We performed immunohistochemistry as described previously^41^. Briefly, male adult mouse brains were fixed in 4% paraformaldehyde and 40-μm sections were cut with a cryostat. For staining with anti-BrdU antibody, brain sections were incubated for 15 min with 2 N HCl. The antibodies used were anti-BrdU (1:1,000, AbD Serotec), anti-DCX (1:500, Abcam), anti-CD68 (1:500, AbD Serotec), anti-Iba1 (1:500, Wako), anti-TLR9 (1:100, Imgenex), anti-GFAP (1:2,000, Millipore), anti-S100β (1:500, Sigma), anti-GFP (1:500, Aves Labs) and anti-NeuN (1:500, Millipore). Nuclei were stained using bisbenzimide H33258 fluorochrome trihydrochloride (Hoechst) (Nacalai Tesque).

### Immunocytochemistry

Cells were fixed in 4% paraformaldehyde and processed for immunostaining as described^41^. Cells were stained with one of the following antibodies: anti-Iba1 (1:500, Abcam), anti-GFAP (1:500, Millipore), anti-active caspase3 (1:500, R&D Systems), anti-BrdU (1:500, AbD Serotec), anti-p65 (1:500, Abcam) and anti-CD11b (1:500, AbD Serotec). For anti-BrdU staining, fixed cells were incubated with 2 N HCl for 5 min. EdU staining was performed using the Click-iT EdU Alexa Fluor 555 Imaging Kit (Life Technologies) according to the supplier’s protocol. Stained cells were visualized with a fluorescence microscope (Zeiss Axiovert 200M, Zeiss).

### Confocal imaging

Fluorescence images were obtained on a confocal laser microscope (LSM710 and LSM780, Zeiss). For quantification of the percentage of NS/PCs contacted by microglia, Z-series stacks of confocal images of GFP-expressing Sox2-positive cells and Iba1-positive cells were taken. At least 50 randomly chosen GFP-positive cells in the DG per animal were analyzed. GFP-positive cells whose cell bodies contacted microglial processes or cell bodies were counted. For analysis of neuronal localization in the DG at 8 days after seizure, Z-series stacks of confocal images of DCX-positive cells with Hoechst staining were taken to determine the localization of DCX-positive cells. For analysis of dendritic complexity and measurement of dendrite length *in vivo*, three-dimensional reconstructions of the entire dendritic processes of individual DCX-positive neurons were made from Z-series stacks of confocal images as previously described^42^. Briefly, images were acquired at 0.5-μm intervals. The projection images were traced and analyzed with ImageJ. At least 10 randomly chosen DCX-positive cells in the DG per animal were analyzed. Sholl analysis for dendritic complexity was performed by counting the number of dendrites that crossed a series of concentric circles at 10-μm intervals from the cell soma.

### Cell counts

BrdU-positive cells within the DG were counted using every 6th section (240 μm apart). The number of counted cells was then multiplied by six to provide an accurate estimation of the number of cells per DG. To calculate the total number of marker-double-positive cells, at least 200 randomly chosen BrdU-positive cells per animal were analyzed. Microscopic analysis yielded a ratio of BrdU-positive cells colabelled with DCX, NeuN, GFAP and S100β. These ratios were multiplied by the total number of BrdU-labelled cells to give estimates of the total number of BrdU-positive immature or mature neurons and BrdU-positive astrocytes. To estimate the ratio of surviving cells after seizure, the total number of BrdU-positive cells at 3 weeks post injection of BrdU was divided by the total number of BrdU-positive cells at day 1 post injection of BrdU.

### Cell culture

We obtained primary microglia and astrocytes from mouse at postnatal day 1 (P1) using a previously described protocol^43^, with some modifications. To obtain mixed glial cell cultures, cortexes of WT and TLR9-KO mice were carefully dissected after stripping of meninges. The tissue was digested with papain (Sigma) at 37 °C for 20 min. After centrifugation (200*g*, 5 min), the cell pellet was resuspended in alpha-Minimum Essential Medium (MEM) with 5% foetal bovine serum (FBS) and 0.6% glucose, and the suspension was passed through a 40-μm Cell Strainer (BD Falcon). After centrifugation (200*g*, 5 min), the cell pellet was resuspended in DMEM containing 10% FBS and a low concentration of GM-SCF (0.1 ng ml^−1^; PeproTech) to enhance microglial proliferation. These mixed glial cells were plated in poly-L-lysine-coated T75 tissue culture flasks. The medium was renewed every 2–3 days. Ten days after plating, microglia and oligodendrocyte precursor cells (OPCs) were detached from astrocyte monolayer sheets by shaking, collected and plated onto uncoated 35-mm culture dishes to remove OPCs. After a 30-min incubation, the medium was removed by suction and DMEM containing 10% FBS without GM-SCF was added to the dish. Two days after plating, primary microglia from WT and TLR9-KO mice were used for assays.

After the shake-off procedure for the isolation of microglia, Trypsin EDTA solution (Nacalai Tesque) was added to the flask to obtain the remaining astrocytes, which were transferred to a 35-mm culture dish and maintained in DMEM containing 10% FBS. Two days after plating, the astrocytes were used for assays.

Neuronal cultures were prepared from P1 mouse hippocampus according to a previously described protocol^44^, with some modification. In brief, the hippocampus was digested with papain at 37 °C for 20 min and triturated with a 1-ml pipette. MEM with 5% FBS and 0.6% glucose was added and the mixture was plated onto a poly-L-lysine-coated 35-mm culture dish. After 3 h, the medium was replaced with maintenance medium (Neurobasal Medium (Gibco) supplemented with B27 (Gibco)) containing cytosine β-D-arabinofuranoside (5 μM; Sigma) to eliminate proliferating cells. To avoid neuronal cell death by a complete medium change, half of the medium was replaced every 3 days with fresh maintenance medium. After 12 days, the neurons were used for assays.

To obtain NS/PCs, P1 mouse hippocampus was dissected and triturated in Hank’s balanced salt solution. After centrifugation (200*g*, 5 min), the cell pellet was resuspended in N2-supplemented DMEM/F-12 medium containing 10 ng ml^−1^ bFGF (PeproTech), plated on a poly-L-ornithine/fibronectin-coated dish and incubated for 4 days. The cells were passaged by replating on an ornithine/fibronectin-coated dish in N2 medium with 10 ng ml^−1^ each of bFGF and EGF (PeproTech)^45^. NS/PCs that had been passaged 10 times were used for qRT–PCR analysis.

### aNS/PC proliferation assay

For the *in vitro* aNS/PC proliferation assay, we used aNS/PCs derived from rat hippocampus as previously described^46^. To determine the effect of candidate cytokines, aNS/PCs were cultured in the presence of murine TNF-α, murine IL-12 and murine IFN-γ (all PeproTech) for 3 days. N2-supplemented DMEM/F-12 medium containing bFGF (5 ng ml^−1^) was used as culture medium in this assay.

### Cell supernatant collection

To analyze the role of microglia in aNS/PC proliferation, microglia obtained from WT mice were cultured in the presence or absence of ODN1585 (0.3 μM; InvivoGen), ODN1826 (1 μM; InvivoGen) and ODN2395 (1 μM; InvivoGen) for 3 h and washed with PBS. DMEM containing 10% FBS was added to microglial cultures and incubated for 21 h. CM was then collected. aNS/PCs were cultured with 30% microglial CM for 3 days. For the aNS/PC proliferation assay, either BrdU or EdU was added (to 10 μM) to the culture medium 30 min before fixation. For TNF-α neutralization experiments, microglial CM was supplemented with 10 μg ml^−1^ anti- mouse TNF-α (R&D Systems) or 10 μg ml^−1^ normal goat IgG as a control and incubated at 37 °C for 1 h before it was added to the aNS/PC culture.

To confirm microglial activation by endogenous ligand derived from neurons, neurons were cultured in the presence or absence of KA (100 μM) for 1 h and washed with Neurobasal Medium. To avoid neuronal cell death caused by a complete medium change, we then added a 1:1 mixture of fresh and used maintenance medium, which is CM from cultured neurons without any stimulation. After 24 h, we collected the CM. Primary microglia were treated with 30% neuronal CM. To determine whether endogenous ligand derived from degenerating neurons was DNA, CM from KA-treated neurons was pre-incubated with 50 μg ml^−1^ of DNase (Roche) at 37 °C for 1 h. For NF-κB activation analysis, primary microglia were treated with 30% CM from unstimulated or KA-stimulated neurons. Microglia cultured with the CM were stained with anti-p65 antibody. The TLR9 agonist ODN1826 was added to primary microglia as a positive control to induce the translocation of p65 protein into the nucleus. To determine whether NF-κB is activated by TLR9 signalling, microglia were pretreated with TLR9 antagonist (ODN2088) for 1 h before addition of CM and the translocation of p65 into the nucleus was examined.

### Osmotic pump infusion

In murine TNF-α-infusion experiments, KA-treated TLR9-KO mice were infused with recombinant TNF-α (120 ng per day; PeproTech) into the right ventricle by osmotic minipumps (Alzet) for 5 days with the following coordinates: posterior=0.34 mm from Bregma, lateral=1 mm, ventral=3 mm, as previously described^47^.

### Evans blue injection

A 2% solution of Evans blue dye (Sigma) in 0.9% NaCl was intravenously injected into WT and TLR9-KO mice in a dose of 2 ml kg^−1^ at 3 days after seizure. All mice were killed 3 h after the Evans blue injection.

### Statistical analysis

Statistical comparisons were made by Student's *t*-test (for two-group comparisons) and analysis of variance (for multiple group comparisons) with Tukey *post-hoc* tests. Data represent mean+s.e.m.

## Author contributions

T.M. contributed to the concept, design, execution and analysis of the experiments, provided funding and wrote the manuscript. N.M. contributed to the design and analysis of the experiments. Y.K. and B.J. contributed to analysis of the experiments. J.K., S.A. and T.K. provided advice and technical expertise. K.N. supervised the project and contributed to the concept and design of the experiments, provided funding and wrote the manuscript.

## Additional information

**How to cite this article:** Matsuda, T. *et al.* TLR9 signalling in microglia attenuates seizure-induced aberrant neurogenesis in the adult hippocampus. *Nat. Commun.* 6:6514 doi: 10.1038/ncomms7514 (2015).

## Supplementary Material

Supplementary Information Supplementary Figures 1-15 and Supplementary Table 1
