# Supplementary material for: TLR9 signalling in microglia attenuates seizure-induced aberrant neurogenesis in the adult hippocampus
Source: Nat Commun. 2015 Mar 9;6:6514. doi: 10.1038/ncomms7514 (PMC4366529; doi:10.1038/ncomms7514)
Supplement: Supplementary Information — Supplementary Figures 1-15 and Supplementary Table 1 [file ncomms7514-s1.pdf]

Supplementary information

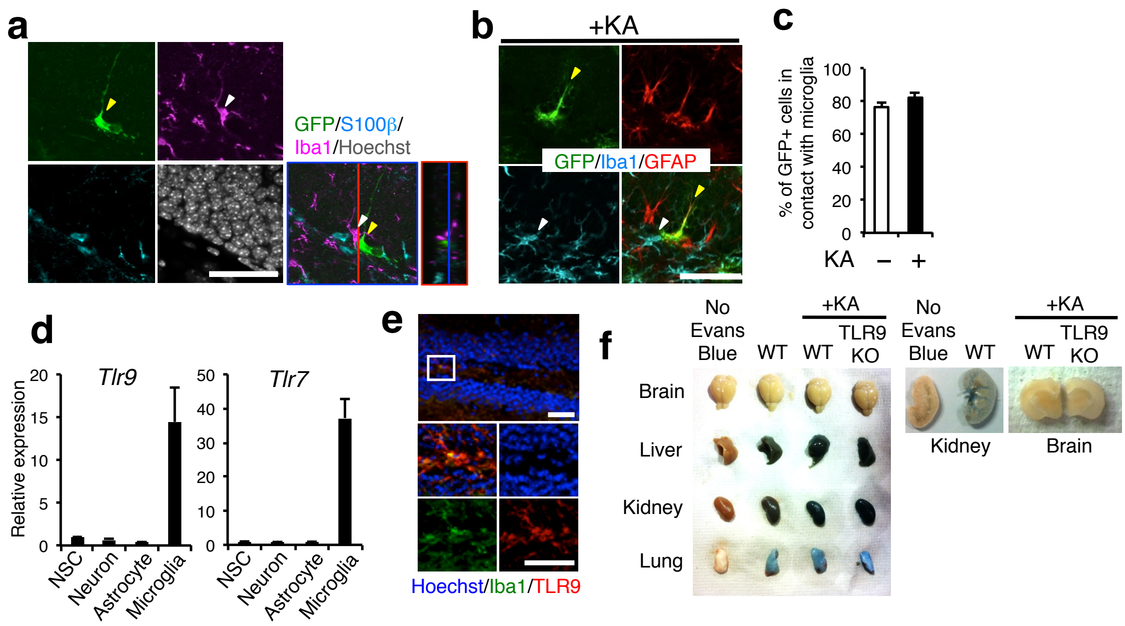

Supplementary Figure 1 The close positioning of TLR9-expressing microglia with aNS/PCs and maintenance of blood brain barrier integrity after seizure induction. (**a,b**) Representative images of GFP, S100β and Iba1 staining in SGZ sections of Sox2-GFP mice under physiological (**a**) and KA-induced pathological (**b**) conditions. S100β+ (blue) astrocytes (**a**), Sox2-GFP+ (green) (**a**, yellow arrowhead) and Sox2-GFP+ (green) GFAP+ (red) (**b**, yellow arrowhead) aNS/PCs are in close contact with Iba1+ microglia (**a**, magenta; **b**, blue) (white arrowheads). Scale bars, 50 µm. (**c**) Quantification of GFP+ aNS/PCs that are in contact with Iba1+ cells under both physiological and KA-induced pathological conditions. (n = 3 experiments). (**d**) qPCR analyses of *Tlr7* and *Tlr9* expression levels in the indicated cell types, normalized to the expression levels in NSCs. (**e**) Representative images of staining for Iba1, TLR9 and Hoechst in the DG. Scale bars, 50 µm (left image) and 25 µm (right panel). (**f**) Visual inspection of the Evans blue administrated mice liver, kidney, lung and brain.

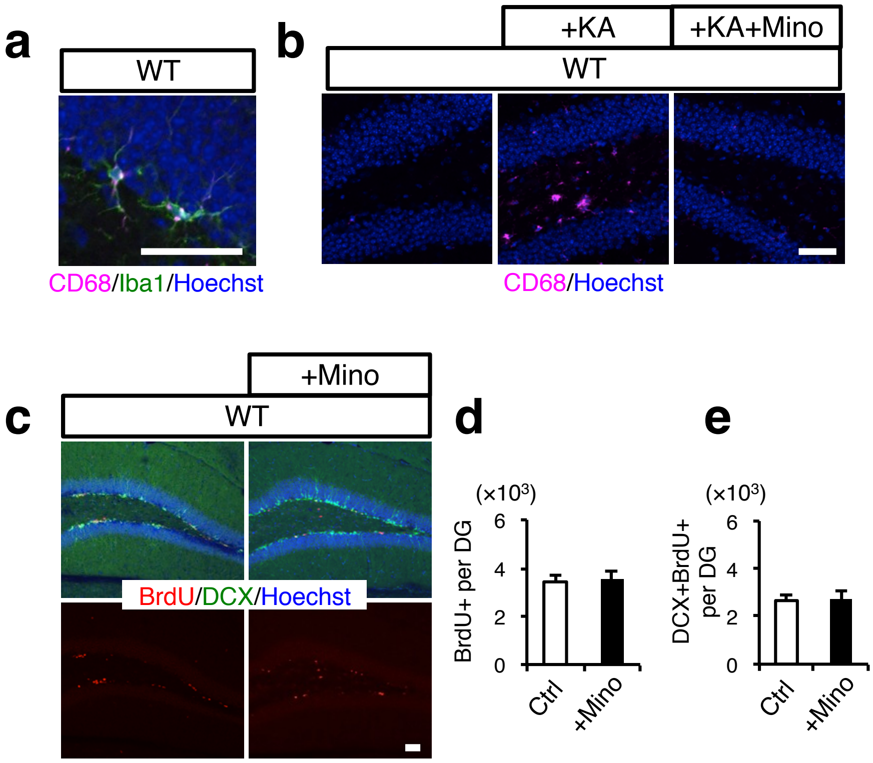

Supplementary Figure 2 Minocycline does not affect neurogenesis under physiological conditions.

(**a**) Representative image of Iba1 (green), CD68 (magenta) and Hoechst (blue) staining in the SGZ of WT mice 7-day after seizure. CD68 was specifically expressed in Iba1+ microglia. Scale bar, 50 µm. (**b**) Representative images of CD68 (magenta) staining in the DG, indicating that seizure-induced microglial activation was inhibited by minocycline treatment. Scale bar, 50 µm. (**c**) Representative images of DCX (green), BrdU (red) and Hoechst (blue) staining in the DG. Scale bar, 50 µm. (**d**,**e**) Quantification of total number of BrdU+ (**d**) or BrdU+DCX+ (**e**) cells in (**c**). There are no differences between minocycline-treated and -untreated mice under physiological conditions (n = 3 experiments).

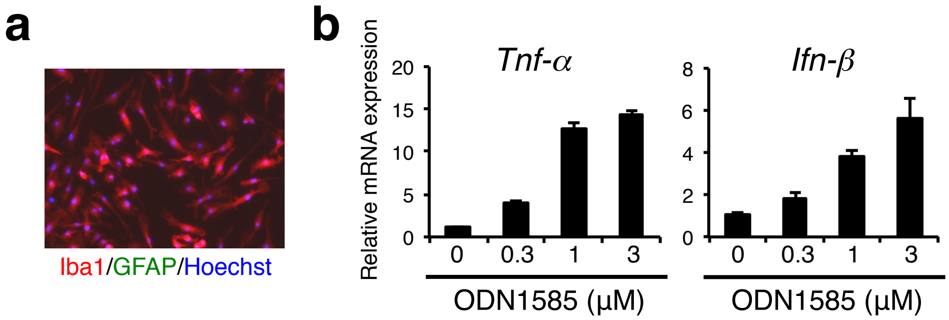

Supplementary Figure 3 The TLR9 ligand ODN1585 activates primary cultured microglia.

(**a**) Primary cultured microglia were stained with Iba1 (red) and GFAP (green) antibodies. Almost all cells are Iba1+ microglia. (**b**) qPCR analyses of expression of the TLR9-downstream genes *Tnf-α* and *Ifn-β* in microglia after ODN1585 stimulation for 24 h. Microglia are activated in this condition.

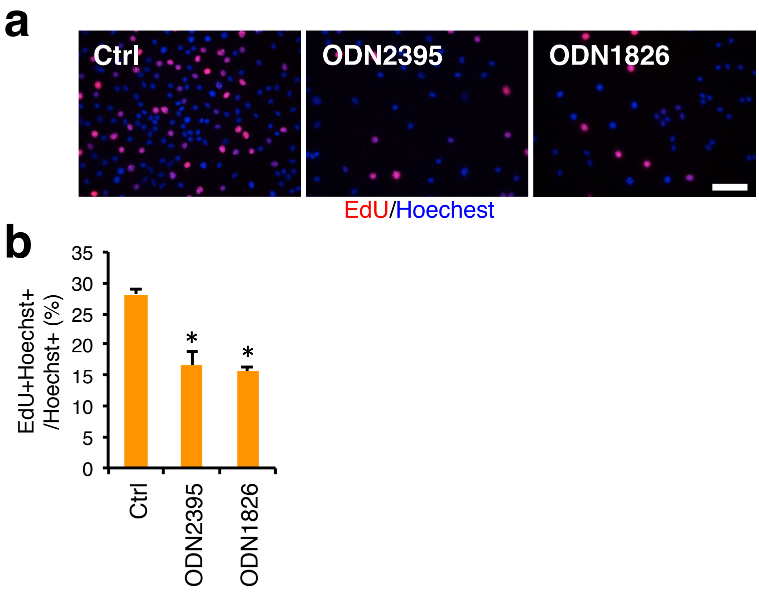

Supplementary Figure 4 Activated microglia by TLR9 ligands inhibit aNS/PC proliferation.

(**a**) Representative images of EdU (red) and Hoechst (blue) staining of aNS/PCs cultured with CM of untreated and ODN1826- or ODN2395-treated microglia. Scale bar, 50 μm. (**b**) Quantification of EdU+Hoechst+ cells in **(a)**. CM of ODN1826 and ODN2395-activated microglia inhibited aNS/PCs proliferation (n = 4 experiments). **P* < 0.01 by ANOVA with Tukey *post hoc* tests.

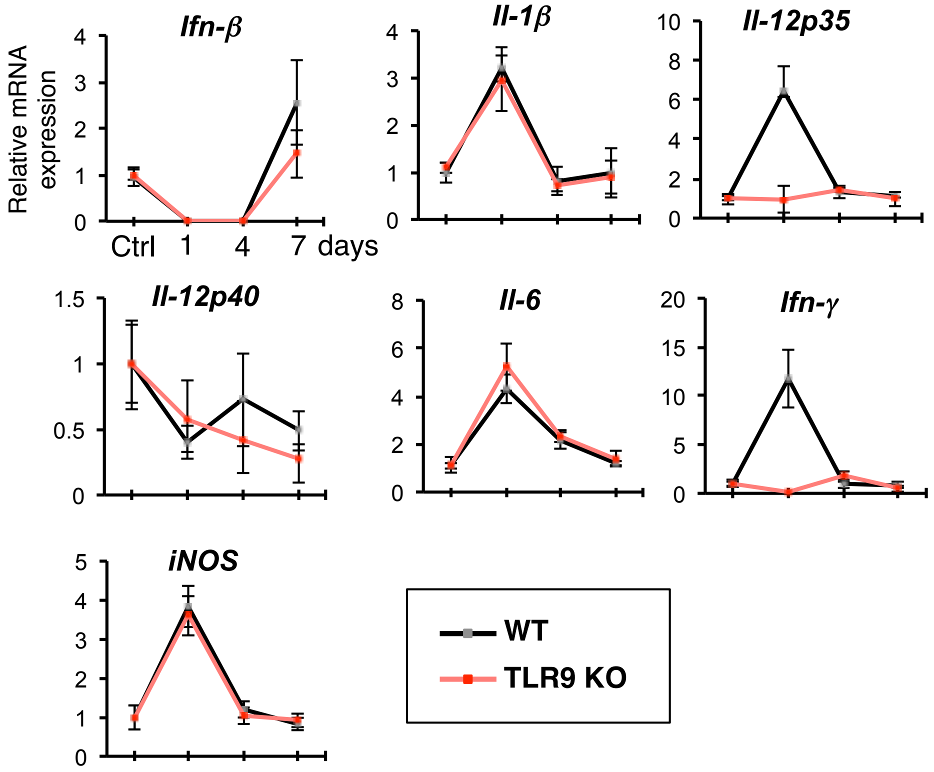

Supplementary Figure 5 Seizure induces expression of inflammation-related genes in the DG.

The graphs show expression analyses of inflammation-related genes in the DG of WT and TLR9-KO mice at the indicated times after seizure induction. Each value was normalized to that observed in corresponding mice without KA-treatment (Ctrl). (n = 3 experiments)

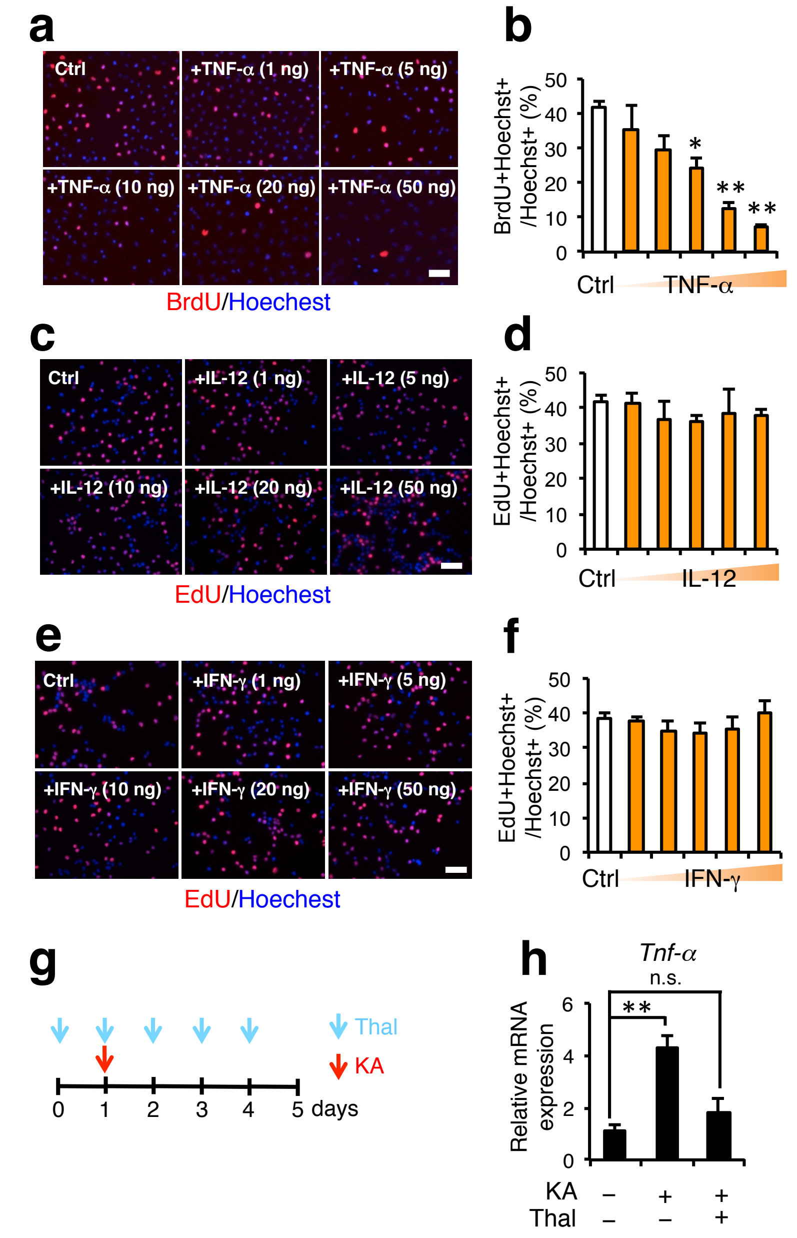

Supplementary Figure 6 TNF-α but not IL-12 and IFN-γ inhibits aNS/PC proliferation in a dose-dependent manner.

(**a, c, e**) Representative images of BrdU or EdU (red) and Hoechst (blue) staining in cultured aNS/PCs with or without stimulation of TNF-α (**a**), IL-12 (**c**) and IFN-γ (**e**). Scale bar, 50 μm. (**b,d,f**) Quantification of BrdU+ or EdU+/Hoechst+ cells in (**a**), (**c**) and (**e**), respectively. (n = 4 experiments). (**g,h**) Seizure-induced *Tnf-α* expression in the DG was decreased in WT mice treated with thalidomide (Thal) (n = 4 experiments). n.s. means not significant ($P > 0.05$). *$P < 0.01$ and **$P < 0.001$ by ANOVA with Tukey *post hoc* tests.

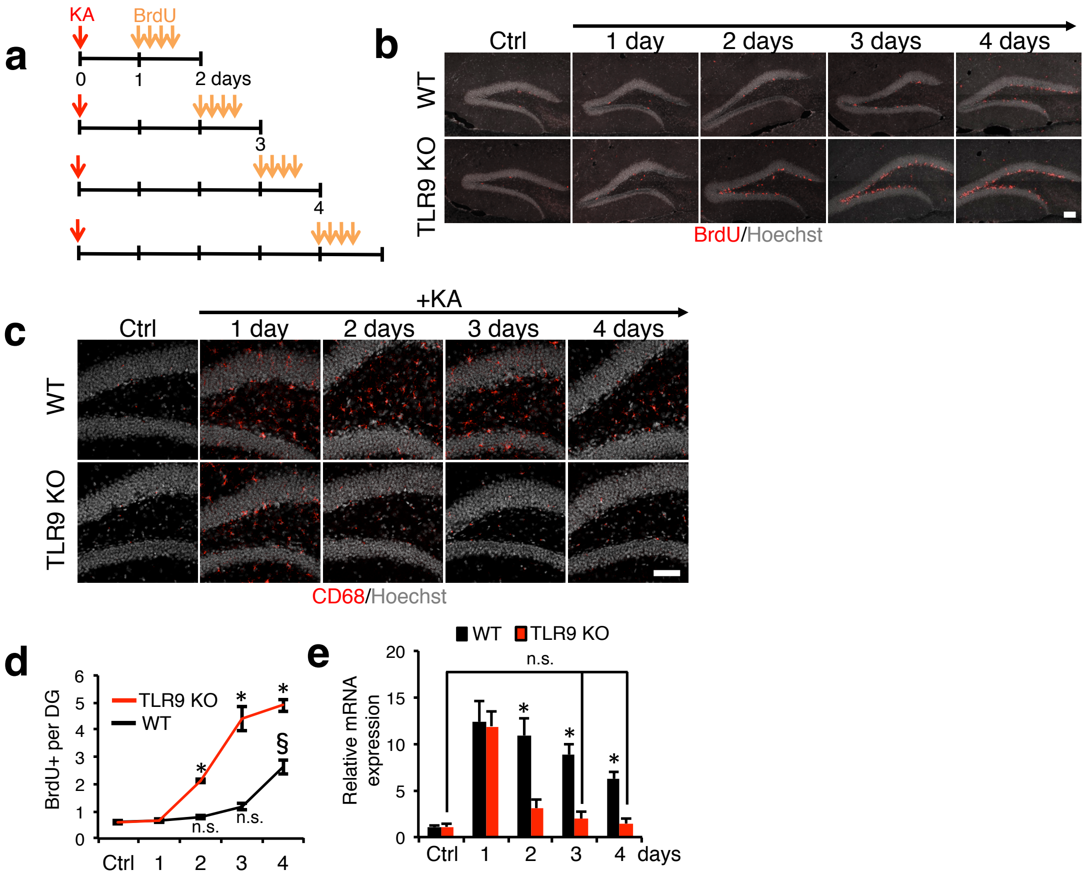

Supplementary Figure 7 Phenotypic differences between WT and TLR9-KO mice start to appear 2-day after seizure induction.

(**a**) Experimental scheme for assessment of aNS/PC proliferation at 1-day, 2-day, 3-day and 4-day after seizure. (**b**) Representative images of the DG stained for BrdU (red) and Hoechst (gray) at the indicated time points after seizure. KA-untreated mice of each mouse strain were employed as controls (Ctrl). Scale bar, 50 μm. (**c**) Representative images of CD68+ (red) activated microglia in the DG of WT and TLR9-KO mice at the indicated time points after seizure. Scale bar, 50 μm. (**d**) Quantification of total number of BrdU-positive (BrdU+) cells within DG in WT and TLR9-KO mice (n = 4 animals). n.s. means not significant ($P > 0.05$). $^{§}P < 0.01$ and $*P < 0.01$ by ANOVA with Tukey *post hoc* tests. (**e**) qPCR analyses of *Tnf-α* expression levels in the DG of WT and TLR9-KO mice at the indicated time points after seizure. Experimental controls were KA-untreated WT and TLR9-KO mice, respectively (n = 3 animals). $*P < 0.01$ by ANOVA with Tukey *post hoc* tests.

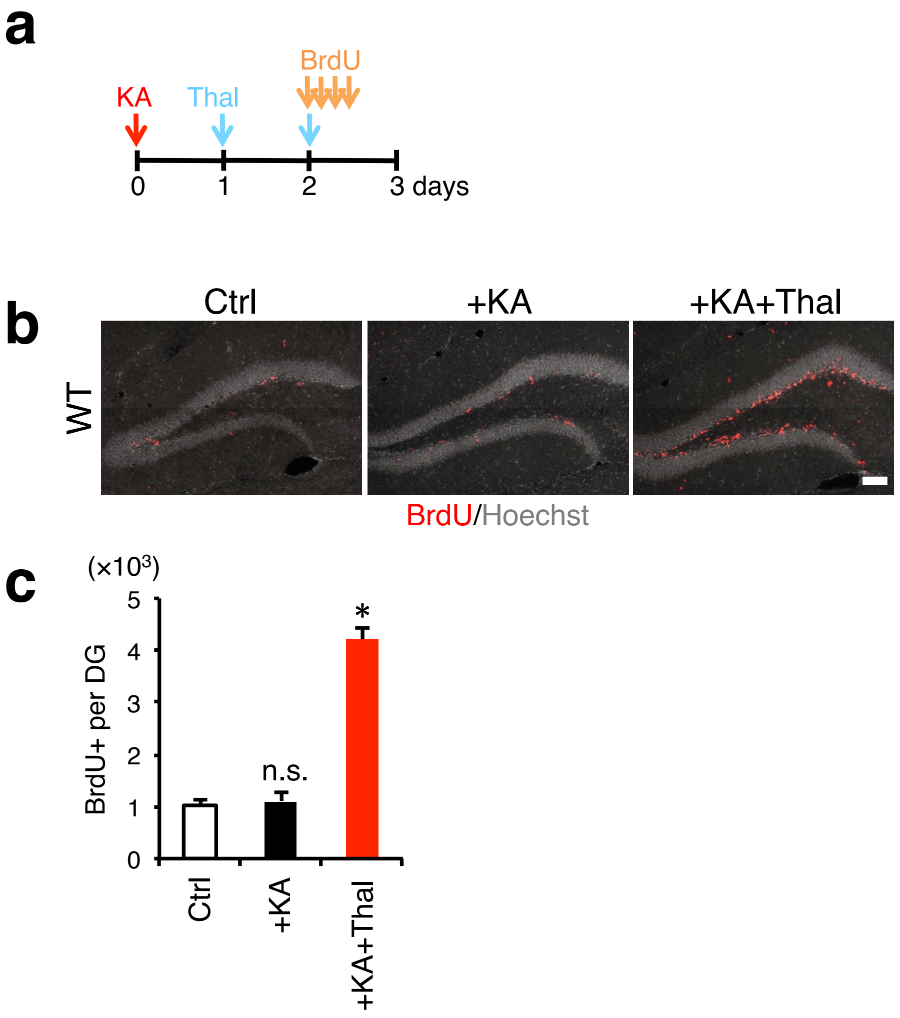

Supplementary Figure 8 Thalidomide exacerbates seizure-induced aNS/PC proliferation.

(**a**) Experimental timeline for assessing aNS/PC proliferation with thalidomide (Thal)-treated WT mice after seizure. Thalidomide was injected 1-day and 2-day after seizure. BrdU was injected at 2-day after KA injection every 4h (4 times) and the mice were sacrificed 12h after the last BrdU injection. (**b**) Representative images of BrdU+ (red) cells in the DG of each treated mouse. Scale bar, 50 µm. (**c**) Quantification of the number of BrdU+ cells in DG (n = 4 animals). n.s. means not significant ($P > 0.05$). *$P < 0.01$ by ANOVA with Tukey *post hoc* tests.

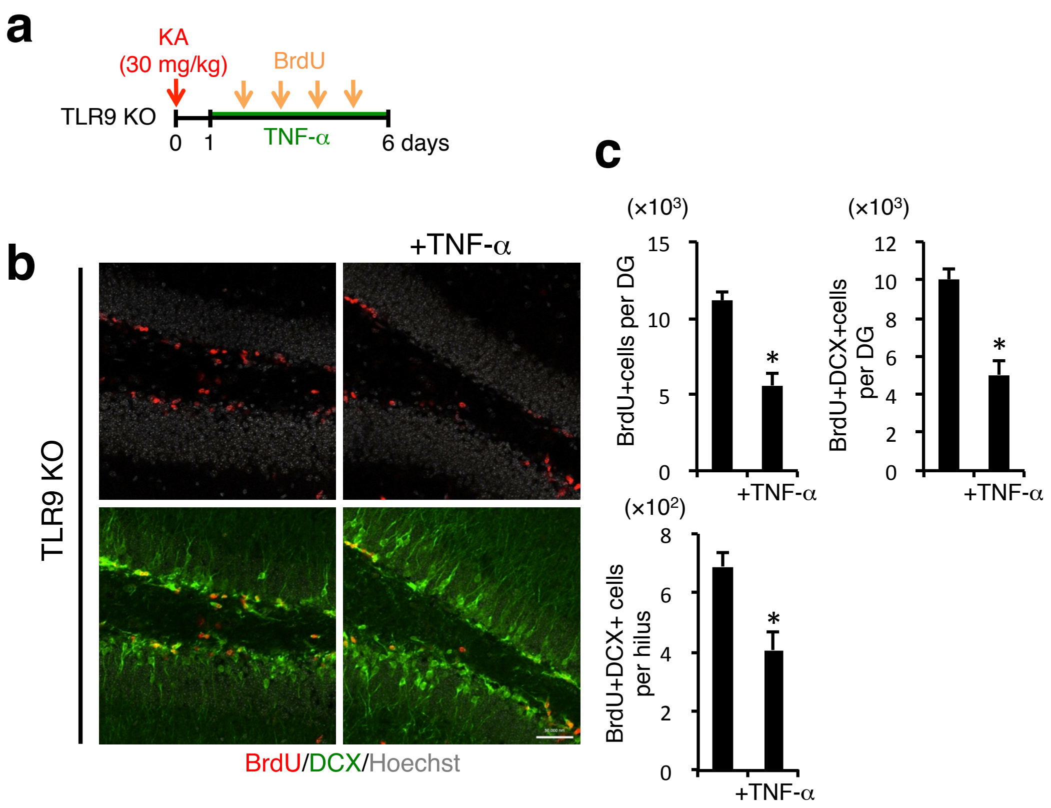

Supplementary Figure 9 TNF-α attenuates seizure-induced aberrant neurogenesis in TLR9-KO mice.

(**a**) Experimental scheme for TNF-α infusion into TLR9-KO mice by osmotic pump (day 1 to day 6 after KA injection). BrdU was injected at day 2, 3, 4 and 5 after seizure. (**b**) Representative images of BrdU (red) and DCX (green) staining in the DG. Scale bars, 50 μm. (**c**) Quantification of the number of BrdU+ or BrdU+DCX+ cells in DG or hilus. The number of BrdU and DCX double-positive newly generated neurons was reduced in both DG and hilus of TNF-α-treated TLR9-KO mice after seizure. *$P < 0.01$ by Student's *t*-test.

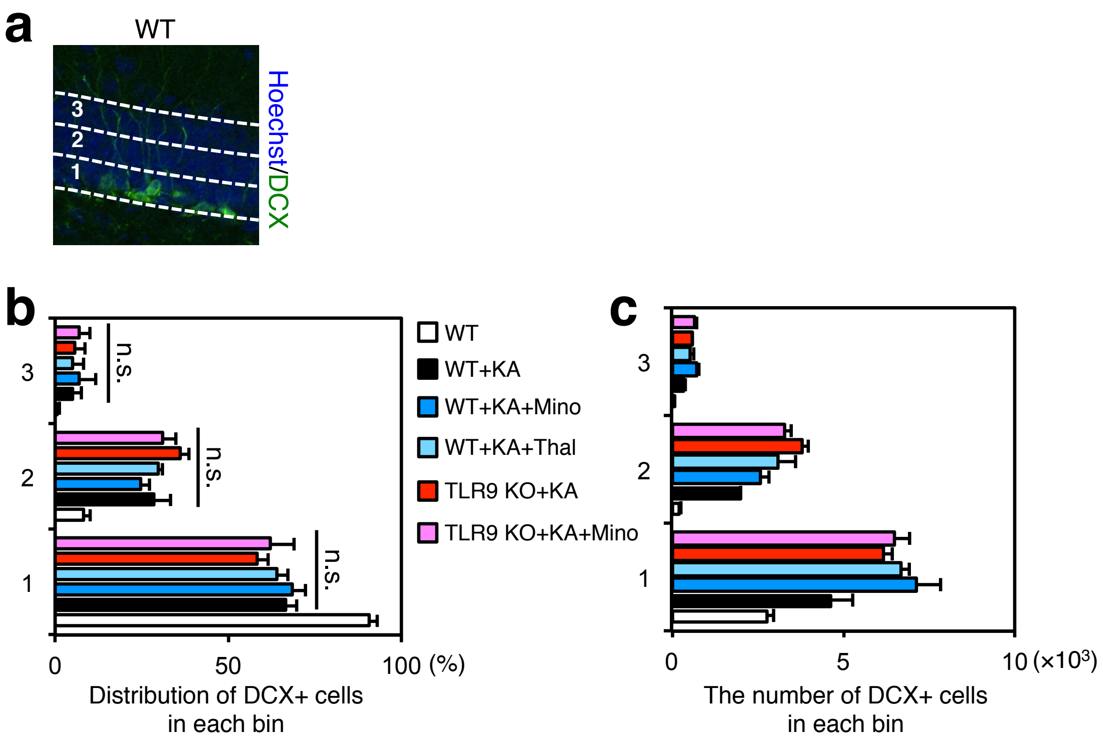

Supplementary Figure 10 TLR9 signaling does not affect the location of DCX-positive cells after seizure.

(**a**) Representative images of DCX (green)-positive neurons in the DG 8-day after seizure. Granular cell layer in the DG was divided into 3 bins and the distribution of the newly generated neurons was analyzed. Hoechst staining indicates nuclei (blue). Scale bar, 50 µm. (**b**) Relative distribution of DCX-labeled cells in each bin divided as in (**a**) was quantified in mice treated as indicated (n = 5 animals). (**c**) The number of DCX+ cells in each bin divided as in (**a**) was counted in mice treated as indicated in (**b**) (n = 5 animals). n.s. means not significant ($P > 0.05$) by ANOVA with Tukey *post hoc* tests.

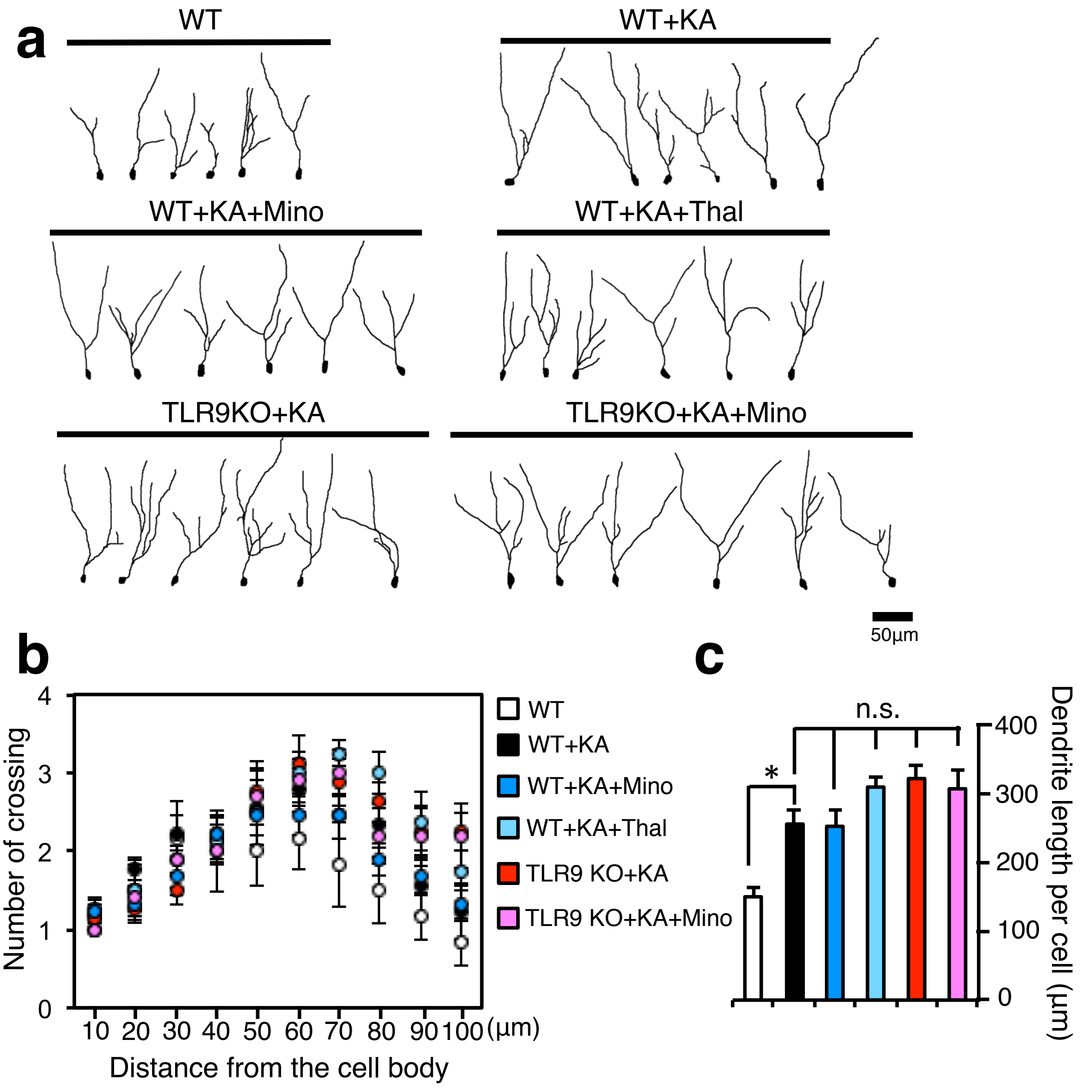

Supplementary Figure 11 TLR9 signaling does not affect the morphology of DCX-positive cells after seizure.

(**a**) Example images of two-dimensional projection trajectories of three-dimensional confocal reconstructions of cell bodies and dendrites of DCX+ cells in DG of each mice as indicated. Mice are treated as in Fig. 3e and 4e. Scale bar, 50 μm. Quantifications of dendritic complexity by Sholl analysis (**b**) and dendritic length (**c**) of DCX-labeled cells were shown (n = 5 animals). n.s. means not significant ($P > 0.05$). *$P < 0.01$ by ANOVA with Tukey *post hoc* tests.

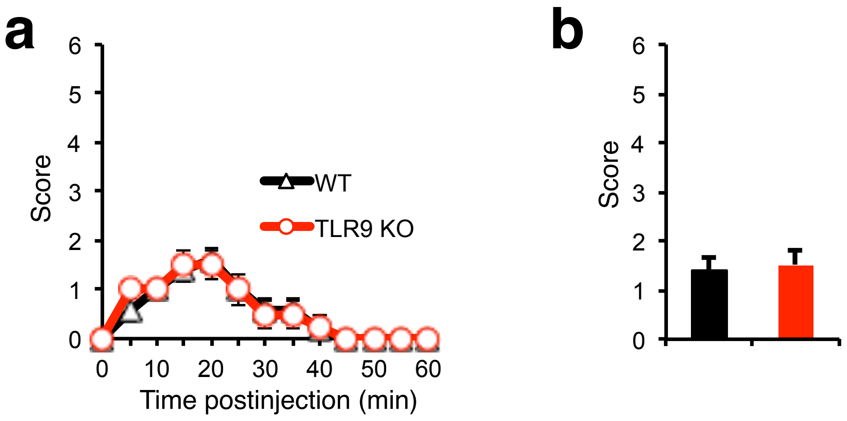

Supplementary Figure 12 TLR9 deficiency does not affect seizure severity.

(**a**) Seizure response over time in WT and TLR9-KO mice following KA injection (10 mg/kg). For each 5-min interval, the highest level of seizure activity was scored using previously described scale (see Methods) (n = 4 animals). (**b**) The maximum score during the 60-min trial in (**a**).

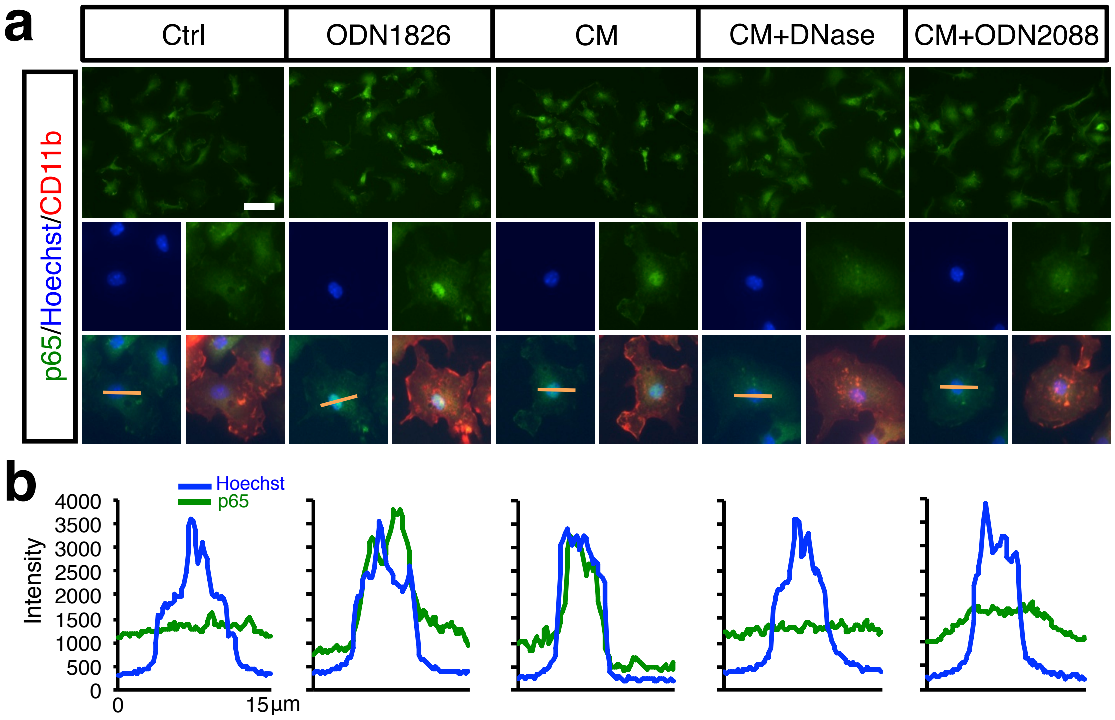

Supplementary Figure 13 DNA derived from KA-stimulated neurons induces NF-κB activation in microglia *via* TLR9

(**a**) Representative images of anti-p65 (green), Hoechst (blue) and anti-CD11b (red) labeled microglia *in vitro*. Microglia were cultured for 1h with CM of KA-treated (CM) or untreated (Ctrl) neurons. ODN1826 (TLR9 ligand) treatment for 1h induced clear nuclear translocation of p65 in microglia. To ablate DNA from CM, CM of KA-treated neurons was incubated with DNase I for 1h. To inhibit TLR9 signaling in microglia, microglia were pretreated for 1h with ODN2088 (TLR9 antagonist) before addition of CM. Scale bars, 50 μm (white) and 15 μm (orange). (**b**) Intensity measurements of fluorescent signals along indicated lines in (**a**) (orange). When p65 is located in the nucleus, the pattern of p65 signal across the nucleus is similar to that of Hoechst, which stains nucleus. This pattern of p65 signal was observed in microglia cultured with ODN1826 and CM of KA-treated neurons, whereas DNase-treatment of CM and ODN2088treatment to microglia abolished this pattern.

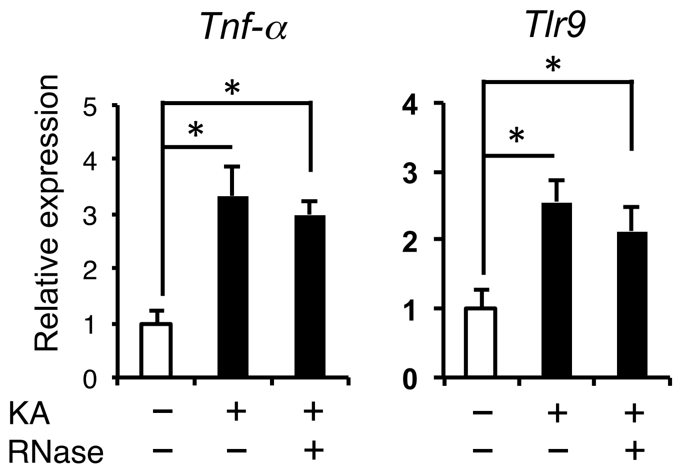

Supplementary Figure 14 RNA derived from degenerating neurons does not activates microglia.

qPCR analyses of *Tnf-α* and *Tlr9* expression levels in microglia from WT mice 12 h after incubation with RNase-pretreated CM from KA-treated neurons (n = 5 experiments). **P* < 0.05 by ANOVA with Tukey *post hoc* tests.

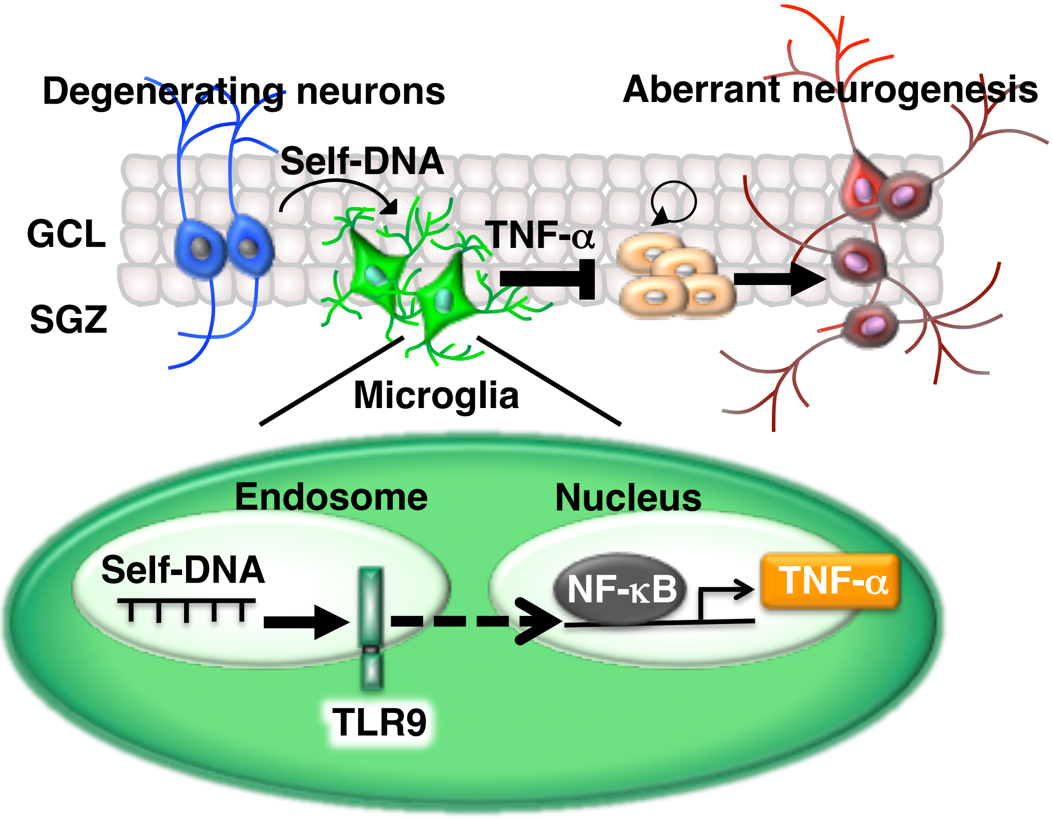

Supplementary Data Figure 15 Schematic representation of the attenuation of aberrant neurogenesis by microglia to maintain homeostatic neurogenesis.

After seizure, aNS/PCs proliferate highly and generate new neurons in ectopic regions, including the hilus. To attenuate this aberrant neurogenesis, TLR9 in microglia recognizes DNA derived from degenerating neurons in the hippocampus and subsequently induces *Tnf-α* expression through NF-κB, resulting in secretion of TNF-α to inhibit aberrant aNS/PC proliferation. GLC, granular cell layer.

Supplementary data Table 1 Primers used for qRT-PCR.

Target gene	Forward primer	Reverse primer
Il-1β	TGAAGTTGACGGACCCCAAA	TGATGTGCTGCTGCGAGATT
Tnf-α	AGGGTCTGGGCCATAGAACT	CCACCACGCTCTTCTGTCTAC
Tlr9	CTGAGCACCCCTGCTTCTA	AGATTAGTCAGCGGCAGGAA
Ifn-β	GCATTTGAAAGGTCAAAGGAA	CCCTTTATAAGAAGTGTCCAGCA
Ifn-γ	TCAAGTGGCATAGATGTGGAAGAA	TGGCTCTGCAGGATTTTCATG
iNOS	GGCAGCCTGTGAGACCTTTG	CATTGGAAGTGAAGCGTTTCG
Il-6	CACAAGTCCGGAGAGGAGAC	TCCACGATTTCCCAGAGAAC
Il-12p35	AAATGAAGCTCTGCATCCTGC	TCACCCTGTTGATGGTCACG
Il-12p40	AGCAGTAGCAGTTCCCCTGA	AGTCCCTTTGGTCCAGTGTG
